# A Systematic Review of Advanced Sensor Technologies for Non-Destructive Testing and Structural Health Monitoring

**DOI:** 10.3390/s23042204

**Published:** 2023-02-15

**Authors:** Sahar Hassani, Ulrike Dackermann

**Affiliations:** Centre for Infrastructure Engineering and Safety, School of Civil and Environmental Engineering, University of New South Wales, Sydney, NSW 2052, Australia

**Keywords:** structural health monitoring, non-destructive testing, non-destructive evaluation, advanced sensor technologies, damage identification methods, machine learning

## Abstract

This paper reviews recent advances in sensor technologies for non-destructive testing (NDT) and structural health monitoring (SHM) of civil structures. The article is motivated by the rapid developments in sensor technologies and data analytics leading to ever-advancing systems for assessing and monitoring structures. Conventional and advanced sensor technologies are systematically reviewed and evaluated in the context of providing input parameters for NDT and SHM systems and for their suitability to determine the health state of structures. The presented sensing technologies and monitoring systems are selected based on their capabilities, reliability, maturity, affordability, popularity, ease of use, resilience, and innovation. A significant focus is placed on evaluating the selected technologies and associated data analytics, highlighting limitations, advantages, and disadvantages. The paper presents sensing techniques such as fiber optics, laser vibrometry, acoustic emission, ultrasonics, thermography, drones, microelectromechanical systems (MEMS), magnetostrictive sensors, and next-generation technologies.

## 1. Introduction

Built infrastructure forms the foundation of human civilization. Ensuring the serviceability and safety of these structures is paramount to our livelihood and the stability and growth of modern society. Routine visual inspections are typically employed to assess the integrity of civil infrastructure, informing engineers and asset owners of any damage or obvious failure of structural components. For bridges, these inspections are completed less than once every two years or on an as-needed basis for other structures such as buildings. For more detailed assessments, visual inspections can be complemented by non-destructive testing (NDT) techniques [1], including eddy-current, ultrasonic, or acoustic emission testing, providing additional information on the properties of a material, component, or system without causing any damage or compromising their functional utility. NDT methods have been developed over many decades and are based on various physical working principles. In 2023 alone, numerous articles have been published on the subject of NDT. For example, Mićić et al. [2] reviewed a wide range of NDT methods for identifying and classifying rolling contact fatigue (RCF) rail defects, mostly squat and head checking (HC) defects. Ramírez et al. [3] compiled a comprehensive state-of-the-art review on NDT techniques applied to additive manufacturing. Furthermore, a review of new developments and applications of ground-based NDT in transport infrastructure monitoring was presented by Gagliardi et al. [4]. Over the last decade, artificial intelligence (AI) has been increasingly used in NDT methods [5]. For example, analyzing ultrasonic signals obtained through NDT tests using machine learning (ML) [6] and deep learning (DL) [7] methods. Shrifan et al. [8] reviewed various NDT techniques for their suitability of applying AI approaches and providing a detailed analysis of AI used for microwave NDT compared to other conventional NDT methods. The application of AI for NDT to Carbon fiber-reinforced polymers (CFRP) was investigated by Schmidt et al. [9]. Their paper presented a quality assurance concept for CFRP prepreg materials and focused on thermographic image classification using convolution neural networks (CNNs). In the paper, an infrared camera and a laser-triangulation sensor were combined to monitor the geometry and impregnation of CFRP prepreg materials. In [10], Liu et al. proposed an NDT method for testing welding defects in seam contours using a laser sensor. DL algorithms were applied for the classification and detection of weld defect images to overcome the problems of low sampling rates and poor recognition accuracy associated with traditional methods.

For continuous structural evaluation, permanently installed structural health monitoring (SHM) technologies [11] provide real-time data on structural parameters and offer many advantages, including cost-effectiveness, instantaneous response to adverse structural changes, improvement of structural reliability, and life cycle management. In general, the main objectives of implementing NDT and SHM techniques are (I) to identify existing and newly developing damage, (II) to locate the damage, (III) to estimate the severity of the damage, (IV) to predict the remaining lifespan of the structure, and (V) to make decisions on structural rehabilitation to extend the lifetime of the structure. Combining SHM methods and AI has recently attracted much attention. For example, Zinno et al. [12] presented an overview of how AI can be used in future data-driven SHM systems. Sujith et al. [13] reviewed smart health monitoring frameworks, which use DL and ML approaches that deliver superior outputs. Interested readers are referred to [14,15] for more information on this subject. In [16], four ML techniques were explored and compared to detect anomalies in monitoring data. The investigated techniques included the image-based time-frequency hybrid convolution neural network (GoogLeNet), the spectrogram-based convolutional neural network, the statistic-based pattern recognition network, the image-based time history convolutional neural network and a proposed ensemble neural network model. All these techniques were found to be successful in detecting and classifying six types of data anomalies (minor, missing, outlier, trend, square, and drift).

Recent years saw the rapid development of sensor technologies, data science, Internet of things (IoT), robotics, and remote sensing, leading to a fast evolution of conventional techniques typically used for NDT and SHM to highly advanced technologies integrated into intelligent sensing systems [11,17,18,19]. Several research examples using new innovations in advanced sensing technologies are presented in Table 1.

Technological advancements, combined with the ever-growing need to maintain our aging infrastructure, resulted in a significant increase in industrial NDT and SHM applications, including the assessment of civil infrastructure, transportation systems, water distribution, and electricity networks [44]. According to a recent report by the IMARC Group [45], the global SHM market is expected to grow from USD 1.7 Billion in 2021 to USD 3.8 Billion by 2027, exhibiting a compound annual growth rate (CAGR) of 14.5% between 2022 and 2027. According to the report, the causes for the growth of the SHM market are as follows: (1) aging infrastructure and superior benefits associated with SHM; (2) loss of lives and capital due to catastrophic failure of infrastructure in recent years; (3) automation and standardization in the maintenance and repair of civil infrastructure; (4) declining cost of SHM systems; (5) increasing capital investments in SHM across various countries worldwide; and (6) stringent government regulations pertaining to the sustainability of structures.

Nowadays, in modern society, NDT and SHM systems are integral parts of various structural systems to maintain safety and reliability, including motor vehicles [46], aircraft [47], pipelines [48], trains [49], refineries, oil platforms [50], bridges [51], and power stations [52]. These techniques are used for different assessments and monitoring needs for civil infrastructure. Table 2 lists various aims for different types of civil structures.

The fast evolution of sensing technologies and data analytics motivated the need for a systematic and comprehensive review of advanced sensor techniques used for NDT and SHM applications. The paper’s extensive literature review presents numerous sensing technologies, from conventional to recently developed next-generation techniques. The review study aims to assist in selecting appropriate sensor technologies for civil infrastructure by providing an overview of available sensing systems, including working principles, sensor design, applications, data transmission, signal processing, and evaluation. Presented sensing technologies and monitoring systems are selected based on their capabilities, reliability, maturity, affordability, popularity, ease of use, resilience, and innovation. Tables are presented as complementary tools to summarize and categorize various advanced sensor technologies and associated systems.

The main contributions of our review paper are addressed in the following:

- We provide a detailed discussion on the relationship between NDT and SHM strategies, elaborating on differences and highlighting similarities.
- We present a comprehensive overview of various established and newly advanced NDT technologies discussing their advantages and disadvantages. We categorize NDT techniques into different groups providing application examples and the latest research studies. We provide detailed explanations of each method’s working principles, technical specifications, and recent innovations.
- We give an overview of SHM systems with details on methods, measurements, monitoring strategies, and technological benefits. We provide comprehensive information on conventional and advanced sensor technologies, including next-generation systems for SHM. We further address technologies for mitigating environmental and operational conditions (EOCs), such as temperature effects and their applications in SHM systems.
- We present recent advances and trends in signal processing, DL, ML, and AI used for NDT and SHM applications.
- Finally, future directions are discussed based on the knowledge acquired from this comprehensive review.

**Figure 1:**
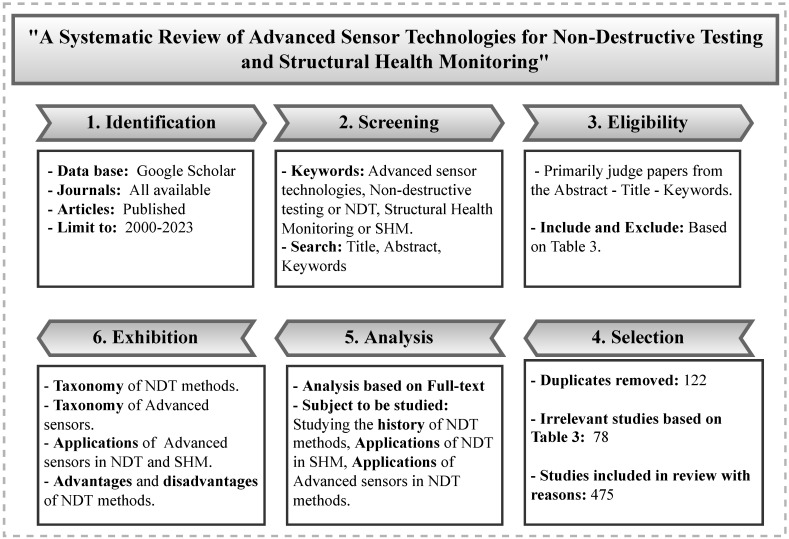
Process for selecting, researching, and analyzing relevant research papers.

**Figure 2:**
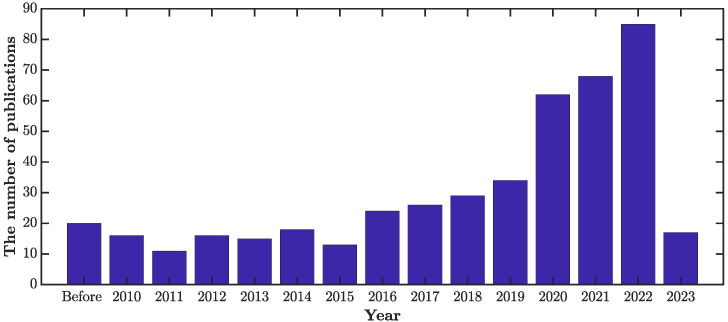
Number of selected and reviewed articles by year.

Choosing the appropriate articles for a review paper is a significant challenge. Figure 1 presents a process flow that guided the various steps of selecting the articles for this literature review. The inclusion and exclusion criteria we chose for the article selection are summarized in Table 3. In total, 475 articles were selected and reviewed. Figure 2 displays a breakdown of these articles by year. As can be seen, over the past decade, there has been a steep incline in research activities in the fields of NDT and SHM, highlighting the rapid developments in this area.

## 2. The Relationship between NDT and SHM Systems

Generally, the relationship between NDT and SHM techniques is fluid and requires an understanding of the definitions of SHM and NDT systems, their historical development, the different types of sensing technologies, data processing methods, assessment aims, and application types. As a short general explanation of the difference between NDT and SHM, the following expression is presented: “NDT typically involves the one-time assessment or short-term monitoring of a material or structural condition, compared to SHM, which involves the permanent installation of sensors on structures for on-going monitoring, while NDT is typically carried out manually or semi-manually by technicians or robots and expert interpretation of sampled data is required, an SHM system can continuously report on the structure’s status, perform the data analysis, and deliver assessment results automatically”.

Historically, NDT methods have been used for centuries, starting with the visual assessment of objects to identify the presence of visible surface flaws to determine their condition. Blacksmiths “listened” to the metal being shaped or the tone of a bell after it was cast. The term NDT was used in 1868 by S.H. Saxby in an article on detecting cracks in gun barrels using magnetic inductions [53]. X-ray techniques were the first industrial applications of NDT methods, followed by magnetic particle crack detection, liquid penetrant testing, eddy current, and ultrasonic testing. In manufacturing, NDT is used to identify potential flaws to be eliminated during production rather than repaired later. NDT is also used to verify that a satisfactory manufacturing process has been achieved. In addition, NDT evaluation may be performed on the component during its life after it has been placed into service. In civil engineering structures, NDT is used in various ways to detect damage, identify material characteristics, and assess structural conditions. NDT techniques are incorporated in regularly scheduled assessment and maintenance programs for the continuous management and monitoring of civil structures and serve as beneficial complementary tools. With the advancements in sensor technology and data transmission systems, traditional NDT methods have been incorporated into continuous real-time structural monitoring systems alongside other techniques that can provide continuous structural assessment, such as strain gauges, accelerometers, or LVDTs. For asset managers to develop viable structure maintenance plans, SHM can provide additional information regarding the structure’s current state and predicted future performance. For structural assessment, various components are considered, including sensing networks, system identification, damage diagnosis, and damage prediction.

NDT and SHM techniques differ in several aspects:

- **Sensor Network [54]**: Sensing techniques used for NDT assessment typically have a high level of technological sophistication. In most cases, NDT techniques use only one type of sensor. It is common for NDT methods to be operated manually and applied following a standardized procedure. In general, NDT techniques are localized in nature, and they require an understanding of the location of the damage a priori. In SHM systems, on the other hand, a variety of sensors is utilized, and data transmission can be automated using a different transmission system, such as wireless communication networks. Large civil engineering structures benefit from SHM methods since they can cover large areas without needing prior knowledge of the damage location.
- **System Identification [55]**: SHM methods can be used to monitor a structure continuously and provide ongoing assessment information on the global status of the structure. Most SHM methods have the ability to incorporate system identification approaches and are appropriate for assessing modal and structural parameters. The reliable implementation of SHM-based system identification for large civil engineering structures is still an active field of research with developments in advanced sensing technology, data transfer systems, and data analytics. NDT techniques are not well suited to system identification since these methods evaluate local structural features rather than global behavior.
- **Damage Diagnosis [56]**: The use of NDT techniques can assist in identifying localized damage, provided that the area and type of damage are known before the damage is identified. Due to their localized nature, these techniques do not require global structural information and analytical models. The damage characteristics are often reliably predicted by NDT techniques using simple computational algorithms. SHM methods, on the other hand, allow for the global identification of structural damage. Identifying damage using SHM methods can be challenging, as they often require detailed structural geometry and material characteristics. A significant amount of computational effort may be required to identify SHM methods. Damage identification algorithms based on SHM are still under development to improve their reliability, accuracy, and efficiency.
- **Damage Prognosis [57]**: To make informed decisions, civil infrastructure management relies on damage prognosis methods that are precise and reliable. NDT methods can deliver accurate damage identification and assessment; however, due to their localized nature, they may not be able to provide reliable information on global damage characteristics and the overall structural system. Further, NDT techniques usually cannot model structural capacity and load conditions stochastically because of their short duration. SHM systems, on the other hand, are able to assess the performance of the entire structural system and provide global damage prognostics. Due to the availability of continuously monitored data, SHM methods can provide information for the stochastic modeling of structural systems for reliability analysis. Thereby, it is possible to determine the time-dependent reliability of the structure based on the evolution of the stochastic models.

Not every sensor technique can be clearly classified as either NDT or SHM technology. As such, ultrasonic testing historically started as a localized NDT method. In recent years, however, advances in ultrasonic transducer technology, sensor networks, communication systems, and data analytics provided for ultrasonic testing technology to be integrated into continuous SHM systems. For this review paper, sensor technologies have been categorized as SHM techniques if they are able to provide measurement data continuously and if it is viable for them to be installed permanently on a structure. The technique or method is classified as NDT technology if these criteria are not given. Some techniques, such as ultrasonic testing used in NDT and SHM applications, are discussed in both categories.

## 3. NDT Systems

NDT is a testing and analysis procedure employed to evaluate the properties of a component, material, or system and to identify damage or defects in a material or structure without causing any physical damage. NDT is also referred to as Non-Destructive Inspection (NDI) [58], Non-Destructive Evaluation (NDE) [59], and Non-Destructive Examination (NDE) [60]. NDT techniques are applied in various fields, including civil, mechanical, aerospace, electrical and systems engineering, medicine, archaeology, and arts. Some application examples are bridges, buildings, tunnels, power plants, dams, motor vehicles, pipelines, and aircraft. The primary purposes of NDT are safety, cost reduction, compliance with standards, accident prevention, failure analysis, and corrective action [61]. Various NDT techniques exist that vary according to the physical working principles of the employed sensor technology. Selecting the most suitable NDT method for a given application requires a broader understanding of each NDT testing and evaluation procedure. Application fields of various NDT techniques are listed in Table 4, and Table 5 presents some new developments in NDT techniques for different applications.

### 3.1. Conventional and Advanced NDT Techniques

Based on the developmental stage and establishment status of various NDT techniques, they can be categorized into two classes: conventional and advanced methods.

#### 3.1.1. Conventional NDT

Conventional NDT techniques have been widely applied for decades, and established codes, standards, and best practices have been developed for several techniques and applications. For civil and mechanical structures, conventional NDT methods are utilized to characterize materials, detect structural damage or identify structural components. Despite their potential, these techniques face challenges such as acoustic coupling, required accessibility to structures, and low signal-to-noise ratios in highly attenuating materials. Examples of conventional NDT techniques are Visual Inspection (VI) [85,86], Infrared Testing (IR) [87], Acoustic Emission Testing (AE) [88], Electromagnetic Testing (ET) [89], Liquid Penetrant Testing (PT) [90,91], Radiographic Testing (RT) [92], Magnetic Particle Testing (MPT) [93], Ultrasonic Testing (UT) [94,95,96], Film Radiography (FR) [97], Eddy Current Testing (ECT) [98], Straight Beam Ultrasonic Testing [99], Leak Testing (LT) [100], and Magnetic Flux Leakage (MFL) [101].

#### 3.1.2. Advanced NDT

Advanced NDT techniques typically employ emerging technologies and involve more complex system setups and data analysis procedures compared to conventional methods. Since these techniques are largely new technological developments, most civil engineering applications are currently still limited to research or small-scale field applications. Due to the novelty of these methods, there is still a knowledge gap with unclear advantages and disadvantages for certain applications and a lack of technical training and practice confidence, while technicians can easily be trained to employ the most advanced methods, the implementation, operation, and analysis of the measured data can be complex and can require specialized training and expertise. Continuous technological advances lead to further developments of each technique, thereby initiating a new revolution in technician training and technological knowledge. An example of an advanced technique is phased array ultrasonic testing (PAUT), whereas straight beam ultrasonic testing (UT) [102] is considered a conventional technology that has matured over many decades and is widely being applied. Other examples of advanced NDT methods are Laser Profilometry [76], Alternating Current Field Measurement [103], Angle Beam [104], Automated Ultrasonic Backscatter Technique (AUBT) [105], Holographic Testing [106], Laser Shearography [107], Computed Tomography (CT) [108], Digital Radiography (DR) [109], Computed Radiography (CR) [110], Electromagnetic Acoustic Transducer (EMAT) [111], Time-of-Flight-Diffraction (TOFD) [112], Immersion Testing [113], Long Range Ultrasonic Testing (LRUT) [114], Internal Rotary Inspection System (IRIS) [115], and Phased Array Ultrasonic Testing (PAUT) [116].

### 3.2. NDT Categories

NDTs comprise a wide range of applications, and many NDT technologies have been developed in recent decades and are based on vastly different physical working principles. They can be grouped based on their primary physical mechanism to provide an overview of the different types. Seven categories of NDT technologies are discussed below:

- **Acoustic Wave Methods [117,118,119,120,121,122]:** In acoustic wave methods, sonic and ultrasonic stress waves are detected and monitored. The stress waves are either passively emitted (such as in Acoustic Emission) or actively imparted (e.g., for Guided Wave Testing). They are typically conducted within the elastic material range in order to detect internal flaws and characterize materials. These methods include (1) Acoustic Emission, (2) Acoustic Wave Methods, (3) Ultrasonic Pulse Velocity (UPV), (4) Ultrasonic Testing, (5) Impact-Echo (IE), (6) Trough Transmission, (7) Spectral Analysis of Surface Waves, (8) Nonlinear Ultrasonic Analysis, (9) Resonant Frequency Methods, (10) Ultrasonic Phased Arrays/Ultrasonic Phase Spectroscopy, (11) Time-of-Flight Diffraction, (12) Non-Contact Ultrasound, (13) Microwave Testing, (14) Acousto Ultrasonic (15) Guided-Wave Testing and (16) Pulse-Echo.
- **Electromagnetic and Magnetic Methods [123,124,125]:** Either transmitted or reflected electromagnetic waves pass through an element during electromagnetic testing. Changes in the material’s properties are reflected in changing electromagnetic behavior and inclusions (such as steel reinforcement), and voids within a component can be detected. No electrical current flows through the element in these tests since the waves are not electrically coupled. These methods include (1) Eddy Current Testing, (2) Magnetic Flux Leakage, (3) Microwave, (4) Remote Field Testing, (5) Near-Field Testing, (6) Magnetic Particle Inspection, and (7) Ground-Penetrating Radar (GPR).
- **Optical Methods [126,127,128]:** Optical NDT (ONDT) techniques were likely the first methods to be used for NDT, in particular visual testing. ONDT methods offer several advantages, such as fast and contactless measurements. The non-contact nature of the measurements ensures that the object’s state is not altered and does not influence the measurement results. Specific quantities (e.g., color or reflectivity) can almost exclusively be measured optically. ONDT techniques can be categorized into two types, passive and active. Passive ONDT methods use measurement methods like ellipsometry, reflectometry, or simple visual inspection to detect defects. In active ONDT, hidden defects are detected using an excitation force, such as heating or mechanical vibration introduced by transducers. Many parameters can affect optical measurements, including the object’s physical properties, material anisotropy, stresses, and temperature. As such, a laser-excited Lamb wave propagating on carbon fiber-reinforced plastic (CFRP) has different damping and velocity depending on the propagating direction [129]. ONDT methods include (1) Visual Inspection, (2) Fibre Optics, (3) Fibre Bragg Grating, (4) Laser Shearography, (5) Infrared Thermography, (6) Digital Image Correlation, (7) Electric Speckle, (8) Optical Coherence Tomography, (9) Endoscopic and (10) Terahertz technology.
- **Radiographic Methods [130,131]:** In radiography, ionizing and non-ionizing radiation is utilized to examine the internal structure of objects. The object absorbs some radiation energy depending on its density and structural composition, and energy differentials can be captured using a detector such as a photographic film or digital detector. These methods include (1) X-ray Testing, (2) Gamma Ray Testing, (3) Neutron Radiography, (4) Radiographic Testing, and (5) Neutron Moisture Gauge Testing.
- **Electrical and Electrochemical Methods [132,133]:** In electrical methods, electricity is applied to an element resulting in a net flow of alternating or direct current throughout the element, while during electrochemical testing, an element’s electrochemical potentials are measured using a reference cell. These methods include (1) Corrosion Current, (2) Electrical Conductivity, (3) Corrosion Half-Cell Potential, (4) Capacitive/Resistive Humidity Sensors, and (5) Alternating Current Field Measurement.
- **Chemical and Mass Transport Methods [134,135,136]:** During chemical testing, a chemical is directly applied to an element, with the chemical response acting as a guide to determine the element’s chemical and mechanical state. Mass transport testing is performed to determine either a specific or crucial transport factor, such as porosity. Coefficients of these testing types include hydraulic conductivity (D’Arcy permeability), diffusivity, sorptivity (water uptake), and vapor diffusion (drying rate). Chemical and mass transport methods include (1) pH Testing, (2) Staining (Alkali-Silica Reaction), (3) Carbonation Depth, (4) Maturity/Thermometry, (5) Chloride Analysis, (6) Liquid Penetrant, (7) Dye Penetrant, (8) Leak Testing, (9) Permeability, (10) Sorption and (11) Absorption Testing.
- **Mechanical Impact Methods [137,138]:** In this type of testing, a mechanical impact is imparted on an element generating a static stress field. Typically, the impact goes beyond the elastic behavior of the material, and its mechanical properties are determined, including the point of failure (strength). These methods include (1) Rebound Method, (2) Penetration Resistance, (3) Pullout Testing, and (4) Break-Off Testing.

**Figure 3:**
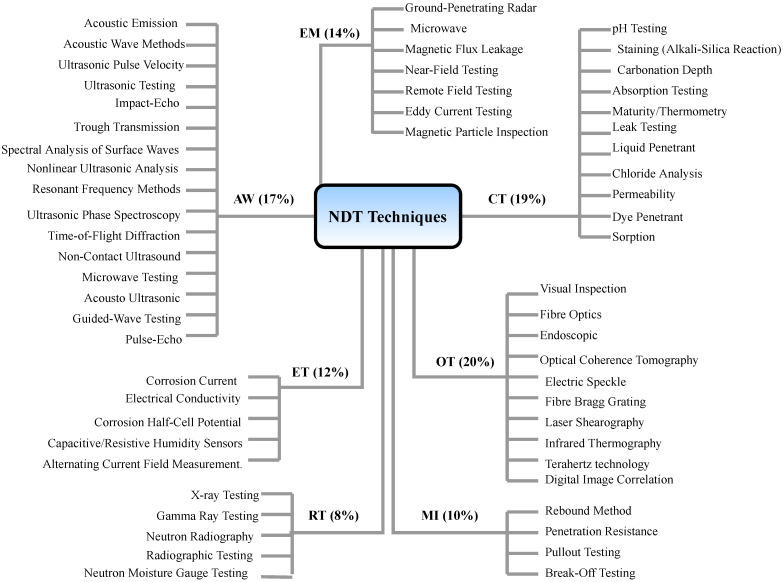
Categories of NDT techniques.

In Figure 3, we provide an overview of the seven NDT categories discussed above (EM: Electromagnetic, AW: Acoustic Wave, RT: Radiographic Techniques, OT: Optical Techniques; ET: Electrical Techniques, CT: Chemical Techniques, MI: Mechanical Impact). This figure shows the number of publications per test over the total number of NDT publications from 2000 to date as a percentage. As can be seen in the figure, CT and OT are the most actively researched NDT methods between 2000 and 2023.

In Table 6, we have listed some inspection types for steel and commonly utilized NDT technologies and probe styles. In Table 7, we present the applications and benefits of common NDT methods used for concrete, wood, composites, and masonry structures.

### 3.3. Sensor Technologies for NDT Systems

For various NDT approaches, different types of sensor technologies, system setups, and data analysis techniques have been developed in recent decades, while some established NDT methods have remained unchanged, others are constantly evolving and improving. The following presents the basic working principles, recent advancements in sensor technologies and system operation, as well as summarized advantages and disadvantages of the various NDT approaches.

#### 3.3.1. Ultrasonic Technologies

**Figure 4:**
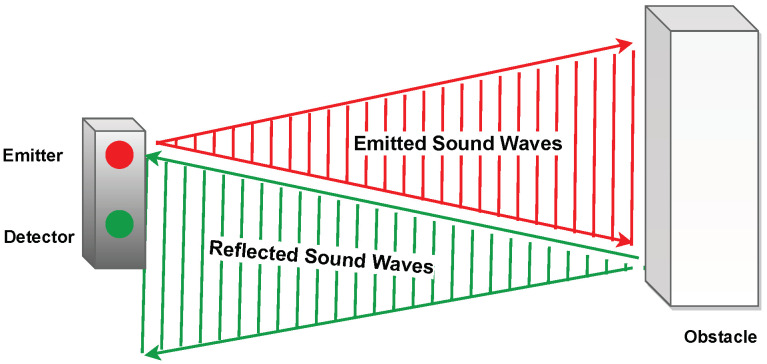
Principle of ultrasonic testing.

In ultrasonic testing (UT), the propagation of ultrasonic stress waves is analyzed to characterize materials and identify internal defects for quality control. Ultrasounds are high-frequency sound waves with frequencies greater than 20 Hz above humans’ maximum hearing threshold. In typical UT, transducers send ultrasonic impulses with frequencies between 0.2 and 15 MHz. For specific applications, frequencies up to 50 MHz are utilized. With increasing ultrasonic frequencies, the wavelength decreases, enabling an increase in measurement accuracy and resolution required for applications such as medical ultrasonography, which uses frequencies of 2–20 MHz [168]. An ultrasonic transducer is an electronic device that can emit and measure ultrasonic sound waves. The sensor converts the reflected sound into an electrical signal allowing the determination of the distance to a target object. Figure 4 depicts the basic operating principles of this technology. Ultrasonic sensors can be categorized into four major types including:

- Ultrasonic Proximity Sensors: An ultrasonic proximity sensor uses a particular type of sonic transducer to transmit and receive sound waves alternately.
- Ultrasonic 2-Point Proximity Switches: This sensor has two switching points and hence has two separate outputs.
- Ultrasonic Retro-reflective Sensors: An ultrasonic retro-reflective sensor works similarly to an ultrasonic proximity sensor, with the difference that it measures the propagation time between the sensor and the reflector.
- Ultrasonic Through Beam Sensors: An ultrasonic through-beam sensor comprises two components: an emitter and a receiver. In the receiver, switching and evaluation outputs are provided.

**Figure 5:**
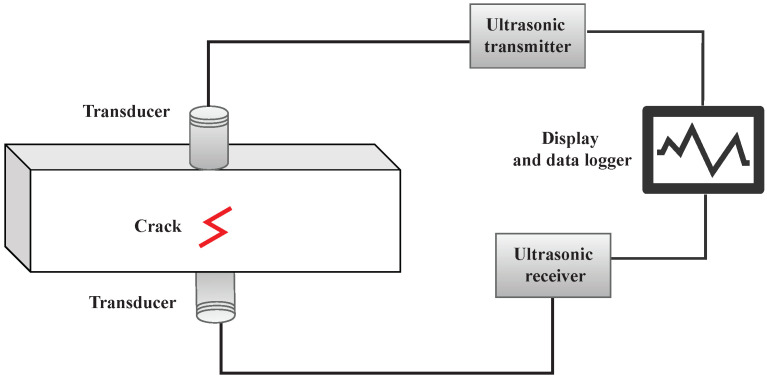
Principle of ultrasonic through-transmission testing.

Typical ultrasonic testing equipment includes ultrasonic transducers, transmitters, receivers, and a data logger. In ultrasonic through-transmission testing, a separate receiver records the propagating ultrasonic waves at a distant site, while in pulse-echo testing, a single transducer sends and receives the pulsed waves. Figure 5 depicts the ultrasonic through transmission testing principle. In the receiving wave signals, wave pattern changes attributed to wave attenuation, reflection, refraction, or energy dissipation effects are analyzed [169]. From the characteristics of these receiving wave signals, such as the wave speed, amplitude or frequency composition, critical information on the examined material can be concluded, e.g., material thickness, discontinuities, temperature differentials, internal defects or material compositions [170]. A comprehensive review paper on this topic can be found in [171] for readers interested in the topic.

In the following, we address the advantages and limitations of ultrasonic testing:

- Advantages: Ability to characterize material properties; can identify, quantify, and localize internal defects; applicable to various materials and components; allows for single-sided assessment; appropriate for assembling lines; fairly affordable; suitable for in situ inspections owing to portable and compact equipment; can detect discontinuities both on the surface and subsurface; long-range inspection capability; minimal preparation requirements.
- Disadvantage: Trained professionals are required to interpret complex wave features and multi-wave modes; limited computing power and algorithms impose resolution restrictions; advanced methods can have complex system setup and transducer designs; highly responsive to environmental and operational changes; damage identification in the vicinity of the transducer probe can be challenging; requires an accessible surface to transmit ultrasound.

Developments in UT have focused on advancements in ultrasonic sensor technologies (e.g., ultrasonic arrays, wireless sensors, sensor sensitivity, signal strength, and wave characteristics) as well as data analysis techniques. For ultrasonic sensors, significant improvements have been achieved over the last few years in terms of accuracy and cost, resulting in more precise, smaller, and cost-effective sensors. An advanced ultrasonic sensor is a one-piece sensor with an outdoor-rated housing. They are typically single ultrasonic transducers that transmit and receive the ultrasonic sound wave. Table 8 lists five types of advanced sensor systems in UT, including self-contained, high-accuracy, close-range, intrinsically safe sensors with accessories, and remote sensing heads.

The following are some examples of essential parameters that cannot be adjusted with basic ultrasonic sensors but can be adjusted by advanced ultrasonic sensors.

- Blanking Distance (or Dead Band): Sets the distance, starting at the sensor face, to the point where the sensor will start measuring target signals.
- Pulses: Controls how many sound waves are sent in each ultrasonic burst.
- Sensitivity: Controls the amplification level applied to a returning signal from the target.
- Temperature Compensation: A correction factor for changes in air temperature.
- Averaging: Defines the number of target samples (readings) that will be averaged to calculate a distance reading.
- Analog Output: Sets the application’s minimum and maximum sensing range.
- Relay Set Points: Sets the minimum and maximum readings desired from the sensor for control purposes.

Sun et al. [172] presented a hybrid ultrasonic sensing system termed diffuse ultrasonic wave (DUW) to detect damage in railway tracks using a lead-zirconate-titanate (PZT) actuator and a fiber Bragg grating (FBG) hybrid sensing system. The experimental results revealed that the DUW signals recorded by the hybrid sensing system have a high potential for damage detection on railway tracks.

Traditional UT cannot be applied for structures with hard-to-reach areas, such as super- or substructures. To address this shortcoming, embedded ultrasonic techniques can be used to detect damage. In [173], Chakraborty et al. presented a methodology for crack detection based on an advanced signal processing algorithm. The method was tested on various reinforced concrete structures, and cracks between embedded sensors were identified. The analysis compared different pairs of embedded ultrasonic sensors for a distance sensitivity study. In an extension of their work, Chakraborty et al. [174] proposed an active method for detecting damage to multiple structures using embedded ultrasonic sensors. Here, raw ultrasonic signals were processed using continuous wavelet transform (CWT) and non-decimated wavelet transform (NDWT) methods to extract damage detection features. In both studies, the researchers confirmed that embedded ultrasonic sensors could monitor real structures more effectively than traditional techniques.

The performance of NDT methods can be adversely affected by high temperatures. Okabe et al. [175] developed an optical fiber ultrasonic sensing system with a phase-shifted fiber Bragg grating (PSFBG) sensor for remote acoustic emission (AE) measurements at high temperatures. The authors incorporated the remote PSFBG ultrasonic sensing system into a laser ultrasonic visual inspector (LUVI) to monitor high temperatures. The results showed that this method can assess the damage progress in heat-resistant materials at high temperatures.

Recently, machine learning algorithms have been applied to analyze ultrasonic signals [176,177]. In [178], ultrasonic test data were used to train six ML models predicting the degree of corrosion in reinforced concrete based on ultrasonic traits. Results showed that ML models could produce accurate and robust predictions of corrosion levels in the presence of outlier amplitudes and for training sets of varying sizes. In [179], Meng et al. proposed a DL-based framework that can be used to classify ultrasonic signals generated from CFRP specimens with voids and delamination. A deep convolutional neural network (CNN) was used in the proposed algorithm to derive wavelet coefficients representing the recorded signals. Compared to classical classifiers with manually generated attributes, the proposed algorithm showed superior performance. A novel damage detection and localization algorithm were proposed by [180] using ultrasonic-guided waves for a composite panel. In the study, two supervised learning-based CNNs were trained on a benchmark dataset to detect damages (binary classifications) and locate them (multiclass classifications). According to the results, the proposed technique was highly accurate (over 99%), computationally efficient (prediction time per signal in milliseconds), provided improved sensor optimization, was robust against noise, and was suitable for in situ monitoring.

Ultrasonic signals can be polluted by Gaussian, speckle, Poisson, or salt and pepper noises, resulting in ultrasonic images that are degraded in resolution and quality. In recent years, DL has been implemented to reduce noise in image and signal data. Singh et al. [181] implemented convolutional autoencoders based on DL to model noise and denoise ultrasonic images. The ultrasonic images were quantitatively analyzed using a structural similarity index measure (SSIM) and peak-signal-to-noise ratio (PSNR) metrics. The authors found that SSIM can measure finer similarities, while PSNR can provide higher visual interpretation.

In the following, we discuss some advanced UT:

- Phased Array Ultrasonic Testing (PAUT): This advanced ultrasonic testing method can be used to inspect welds, assess corrosion, measure thickness, monitor structures, inspect rolling stock (wheels and axles), and perform medical imaging. A phased array ultrasonic scanning unit contains crystals arranged in a pattern to send sound waves in different directions in a material. The technology offers the following advantages: (1) The inspection is fast since no manual sensor movements are required; (2) defect detection is more accurate since a single probe scans all directions simultaneously, reducing additive errors from using multiple angle probes; (3) it provides multiple views of defects using advanced presentation views, such as C-scan view (top view), B-scan view (depth view), S-scan view (sectorial view), and conventional A-scan view (echo pattern). Consequently, the technology is considered a reliable inspection process. An evolution of the PAUT technique is full matrix capture (FMC) which uses the same probes. This method has the advantage of not requiring focusing or steering since the entire area of interest is focused. In addition, it is relatively tolerant of structural noise and misaligned flaws, making it very simple to set up and use. In contrast with conventional PAUT, the recorded data files are huge, resulting in slower acquisition speeds. To improve and automate the data analysis, the capabilities of using machine learning models machine on FMC data were investigated by Siljama et al. [182]. The researchers compared the model performance to the results of a human inspector using real thermal fatigue cracks with similar weld geometries used in training. The results showed a high flaw detection performance using the automated machine learning approach.
- Time of Flight Diffraction Ultrasonic Testing (TOFD): A TOFD system consists of two ultrasonic probes, a “transmitter” and a “receiver” attached to a fixture. There are two types of fixtures: manually operated and robotically operated. This technology detects defects by analyzing the time of wave travel and the diffraction of the wave from crack tips. Hence the name of the test, “Time of Flight Diffraction”. The TOFD technique offers the following advantages: (1) Reliability and reproducibility of inspection; (2) accurate sizing of the depth of the tips; (3) easy storage of the results; (4) quick references and comparisons; (5) monitoring of defect propagation.
- Long Range Ultrasonic Testing (LRUT): This method, also known as guided wave ultrasonic testing, is a cost-effective and fast technique to inspect long pipelines. Using this technology, the complete length of a pipe can be assessed for internal corrosion, damage, or cracks. In LRUT, multiple probes are positioned around the pipe’s circumference, and waves are transmitted by traveling through the pipe wall. Using low-frequency sound waves will prevent the loss of sound due to scattering. A-symmetrical sound echoes indicate locations of defects, and their size can be determined through the distance amplitude curve (DAC). LRUT has the following significant advantages: (1) It is a fast and flexible testing method; (2) an extended length of pipe can be assessed at once; (3) it can scan insulated and underground pipes without the need for excavation.
- Laser Ultrasonic Testing (LUT): LUT uses lasers to generate and detect ultrasonic pulses for non-contact ultrasonic measurements. Sound pulses are generated by short-pulse, high-energy lasers. Due to the lack of physical contact, this ultrasonic technology can be applied to materials of any temperature. As a result, it is ideal for the in situ assessment of solid metallic and ceramic materials up to their melting point. This technology has the following benefits: (1) Remote and non-contact testing, enabling the inspection of samples at shallow and very high temperatures, for example, during welding with restricted access; (2) small and adjustable footprint; (3) allows the inspection of small and complex geometries; (4) uses high wave frequencies to detect microscopic flaws. Lv et al. [6] developed an automatic non-contact LUT to quantify the depth and width of subsurface defects on metallic components using ML algorithms. The objective of this study was to present and compare three widely used machine learning models in NDE, namely, extreme gradient boosting (XGBboost), adaptive boosting (Adaboost), and support vector machines (SVM), in combination with principal component analysis (PCA) for detecting both the depth and width of subsurface defects. The PCA-XGBoost analysis of laser-ultrasonic signals achieved the highest recognition rate, 98.48%, and was therefore found to be the most effective approach.

In Table 9, we review some recent papers using advanced UT, including contactless sensing techniques based on lasers for various applications such as railway track or pipeline monitoring.

#### 3.3.2. Electromagnetic and Magnetic Technologies

In electromagnetic and magnetic testing, electric currents and/or magnetic fields are applied to a test object, and the response is monitored to detect and characterize defects on surfaces and sub-surfaces of conductive materials [193,194]. Since defects cause changes in the electrical conductivity and magnetic permeability in a test object, e.g., changes to flow pattern, intensity, and phase, they can be utilized for their identification. Research in magnetic fields has gained traction in various fields over the past few years. However, the rapid generation of strong magnetic fields has remained a challenge. According to Sederberg et al. [195], with moderate laser intensity, magnetic fields exceeding 8 Tesla can be activated within 50 femtoseconds using an all-optical approach. A comprehensive review paper on this topic can be found in [196] for readers interested in the topic. Electromagnetic and magnetic testing includes Alternating Current Field Measurement (ACFM), Eddy Current Testing (ECT), Magnetic Flux Leakage (MFL), Remote Field Testing (RFT), Magnetic Barkhausen Noise (MBN), Electromagnetic Ultrasonic Guided Wave Testing (EUGW), Metal Magnetic Memory (MMM), Alternating Current Field Measurement (ACFM), Tangential Eddy Current (TEC), Pulsed Eddy Current Testing, Eddy Current Array (ECA), Remote-Field Eddy Current Testing, Low-Frequency Eddy Current Testing, and Electromagnetic Acoustic Transducer (EMAT).

The following addresses the advantages and limitations of electromagnetic and magnetic testing:

- Advantages: Fast; contactless; sensitive to surface defects; detection through several layers and through surface coatings possible; portable; sensitive to small discontinuities; accurate conductivity measurements; can be automated
- Disadvantages: Limited to electrically conductive materials; very susceptible to magnetic permeability changes; unable to detect defects parallel to surface; not suitable for complex geometries or large areas

Magnetic sensors, used for electromagnetic and magnetic testing, can detect magnetism generated by a magnet or current. These sensors convert the magnitude and variation of the magnetic field into electric signals. Two properties are critical in the evolution of magnetic sensors: sensitivity and selectivity. Advanced magnetic systems offer not only high magnetic sensing performance but also reliability, small footprint, high-frequency operation, galvanic and thermal isolation, and low-power consumption or even self-powered operation across a broad spectrum of applications.

In general, there are two types of magnetic sensors: Magnetic position sensors and magnetic speed sensors.

- Magnetic position sensors: These types can detect magnetic fields and identify an object’s positional data. Sensors include Magnetic switches, Linear sensors, Angle sensors, and 3D magnetic sensors.
- Magnetic speed sensors: These sensors measure the speed (and direction, if required) of rotating targets. Examples are Crank speed sensors, Cam speed sensors, Wheel speed sensors, TLE5549 (new TMR magnetic sensor for autonomous parking), and Transmission speed sensors.

To date, a wide variety of magnetic sensors have been developed. Table 10 presents a selection of advanced magnetic sensors currently used in the area of NDT, including superconducting quantum interference devices (SQUID), fluxgates, Hall sensors, anisotropic or giant magneto-resistors (AMR and GMR), Micro SQUID, Nano SQUIDs, Optically pumped magnetometers (OPMs), and Giant magneto-impedance (GMI).

Eddy Current Testing (ECT) is one of the earliest electromagnetic testing methods used in NDT. The principle of ECT is based on the relationship between electricity and magnetism (electromagnetism). ECT consists of three essential components: a flaw detector, a probe (test coil housing), and operating software. The test coil consists of a tightly wound wire (coil) configured in various arrangements. ECT works based on Oersted’s theory of electromagnetism, in which a magnetic field develops from an electric current running through a conductor. Further, ECT follows Faraday’s law of electromagnetic induction, which states that the relative motion between a conductor and a magnetic field causes voltage in the conductor. In ECT, a signal is recorded if the energized coil makes contact with a conductive material and the electrical impedance of the coil changes. ECT instruments measure the change in impedance amplitude and phase angle when the coil passes over flaws or indications, giving instant feedback to qualified inspectors. The technology offers the following advantages: (1) Sensitive to small cracks; (2) ECT equipment is portable; (3) Test probe does not need to be in contact with the testing specimen; (4) Conductive materials with complex shapes and sizes can be inspected using ECT.

Over the past decades, several advanced electromagnetic and magnetic techniques have been developed, enhancing, among other features, the technology’s sensitivity and robustness to environmental effects. As such, interventional electromagnetic thermography is an advanced technique that can reduce the influence of non-uniform emission on subsurface defects in low-emissivity metal materials. Miao et al. [206] proposed an interventional electromagnetic thermography technique for detecting subsurface defects based on radiation parameters and the thermophysical properties of the participating medium. It was demonstrated that the method was superior for detecting natural subsurface cracks. Wang et al. [207] proposed improved magnetic dipole models for quantitatively evaluating thermal effects on magnetic flux leakage (MFL) under both direct and combined effects of temperature and thermal stress. The proposed models were able to size the defects at high temperatures accurately.

In the following, we discuss some conventional and advanced electromagnetic and magnetic technologies:

- Magnetic Flux Leakage (MFL): This inspection aims to detect flaws and material degradation in steel components and structures using electromagnetism. In MFL, magnets are used to magnetize a part temporarily, and if flaws are present, the magnetic field created will show distortions, indicating pitting, corrosion, and wall loss on the part. The technology offers the following advantages: (1) A quick and easy way to detect corrosion in ferromagnetic materials; (2) sensitive to pits of corrosion; (3) suitable for finned tube inspections.
- Tangential Eddy Current (TEC): Another magnetic induction-based inspection technique. Tangential ECT differs from conventional ECT in that the coils are oriented tangentially to the surface. The technology offers the following advantages: (1) Weld cap scanning is possible with the probe design; (2) Crack detection and characterization in carbon steel; (3) Multi-element array probes can cover an extensive area in a short period; (4) Surface preparation or coupling is not required.
- Eddy Current Array (ECA): This method represents an evolution of conventional ECT. By using multiplexed arrays of coils arranged in rows, this method is superior to ECT technology (which uses nothing more than one or two coils). The technology offers the following advantages: (1) Maintaining a high resolution while scanning a large area at once; (2) Reduced requirements for complex robotics to move the probe; (3) A more accurate flaw detection due to C-scan imaging; and (4) Complex shape inspection.
- Pulsed Eddy Current Testing (PEC): This inspection is based on the magnetic field penetration through several layers of insulation or coating to induce eddy currents on the surface of a material. Generally, PEC is used to measure the thickness or detect corrosion on ferrous materials that are covered with insulating fireproofing layers or coatings. Applications include corrosion under fireproofing (CUF), corrosion detection under insulation (CUI), and corrosion blisters and scabs. The technology offers the following advantages: (1) During an inspection; there is no need to shut down the equipment; (2) Insulation material is not altered during inspection; (3) No contact is required between the probe and the component under examination; (4) Surface preparation is not necessary; (5) Component thickness is measured.
- Remote-Field Eddy Current Testing (RFEC): This electromagnetic inspection is particularly suitable for examining ferromagnetic tubes from the inside. In order to generate eddy currents within a tube wall, an exciter coil generates a magnetic field on the inside. By appropriate frequency selection, the depth of the skin equals the thickness of the wall; therefore, eddy currents are generated throughout the wall. The technology offers the following advantages: (1) No contact with the test subject is required; (2) Coverage of a large area; (3) It is highly sensitive to variations in wall thickness; (4) Flexible and portable probe.
- Electro-Magnetic Acoustic Transducer (EMAT): An EMAT generates ultrasonic waves into a test object through electromagnetic induction with two interacting magnetic fields. The Lorentz force can be generated similarly to an electric motor by interacting a relatively high-frequency field produced by electrical coils with a low-frequency or static field produced by magnets. This disturbance induces elastic waves in the material’s lattice. The interaction of elastic waves with a magnetic field induces currents in the receiving EMAT coil circuit in a reciprocal process. When magnetostriction is present in ferromagnetic conductors, it produces additional stresses that can enhance the signals much more than can be achieved via Lorentz force alone. They are used in various applications, such as the inspection of in-service piping, the inspection of tubular, and the inspection of vessels. The technology offers the following advantages: (1) Capacity for dry inspections; (2) Imperviousness to surface conditions; (3) Unique wave modes such as shear waves with horizontal polarization.

In Table 11, we review recent papers using advanced electromagnetic and magnetic technologies.

#### 3.3.3. Shearography Technologies

**Figure 6:**
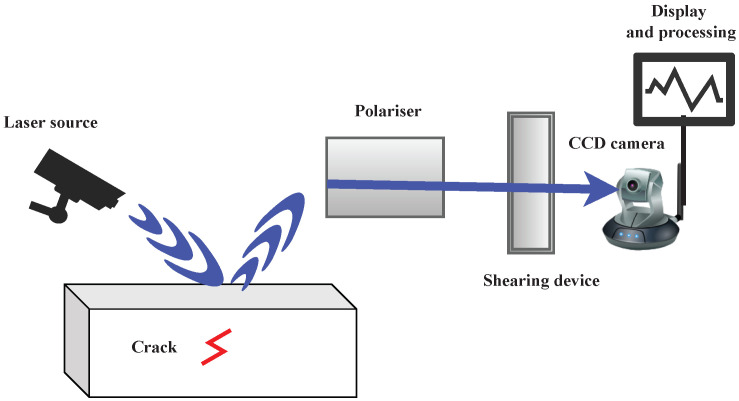
Schematic illustration of a shearography test.

Shearography, also termed speckle pattern shearing interferometry, is a non-contact technique for assessing the quality of materials using coherent light and sound waves for various applications, including wind turbines and aircraft [107]. For example, shearography is currently used on aircraft applications, including the F-22, F-35, Airbus, Cessna Citation X, Raytheon Premier I, and the NASA Space Shuttle. Figure 6 displays the schematic of the working principle of a shearography test. A typical shearography setup includes a polarizer, laser source, CCD camera, and data processor. Shearography instruments and systems have improved dramatically with the development of digital CCD cameras, computer technology, and small, high-power solid-state lasers [214]. As such, digital shearography has become an industry standard in direct strain measurement and for detecting damage to composite materials, such as carbon fiber-reinforced plastics and honeycombs [215]. A comprehensive review paper on shearography technology and applications can be found in [216] for readers interested in the topic.

In shearography testing, a camera or a series of cameras is used to measure the interferometric properties of a material’s surface, utilizing the monochromatic and coherent characteristics of laser light to create an image of the surface. In the first step, a picture of the surface is taken in a neutral or unloaded state. If the surface is not entirely smooth, light reflected from the surface produces a speckle pattern that is recorded by the camera. After the first image is captured, a mechanical load or thermal heating stress is applied to the material resulting in the expansion of any present defects. Next, a second interferometric photograph is taken, capturing the newly deformed and loaded sample with different speckle patterns. By subtracting the second image from the first, a shearogram fringe pattern is produced. This shearogram reveals the topography of any surface defects, including cracking, delamination, debonding, porosity, fluid ingress, and wrinkling. Black and white fringe patterns further provide information about the relative deformation of an object. In a regular pattern, no defects are present; however, in an irregular pattern, the subsurface contains defects. Hence, operators can use shearography techniques to identify defects and measure deformation in materials such as metallic and composite materials. A review of recent papers using the shearography NDT techniques can be found in Table 12.

In comparison to traditional NDT techniques, shearography techniques offer several advantages but also disadvantages:

- Advantages: Non-contact method; suitable for large areas of structures; relatively low sensitivity to environmental variations; ideally suited for honeycomb structures.
- Disadvantages: Possibility of damaging the test specimen, only suitable for specimens with rough surfaces (surface roughness larger than one wavelength of light), requires adequate lightning.

Frequently used shearography NDT techniques include the following:

- Laser shearography: This optical surface measurement technique is based on laser-speckle shearing interferometry [217].
- Vacuum shearography: This method is highly effective in diagnosing delamination, core damage, disbonding, and splice joint separations in composite materials [214].
- Thermal pulse shearography: This inspection technique is used for non-visible impact and pressure damage in composite-wrapped pressure vessels [218].
- Vibration shearography: A novel technique recently developed for NASA’s space shuttle’s external tank foam [219].

Many authors have reported that shearography can be combined with other techniques, such as electronic speckle pattern interferometry (ESPI). This allows the measurement of surface displacements and displacement derivatives simultaneously from a single loading event. Hung et al. [220] used large image shearing to produce ESPI measurements using the reflection of a static surface in a reference beam. For conventional shearography measurements, the same instrument was used with smaller image shearing. Fomitchov et al. [221] developed a dual-purpose system that can be used for ESPI and shearography. The ESPI and shearography functions can be switched using a sliding mirror in the imaging head. Groves et al. [222] combined shearography with digital speckle photography to characterize surface strain. Recent results presented by Rosso et al. [223] demonstrated the feasibility of a combined holography and shearography arrangement that allowed strain and coherent imaging to be measured at the same time. This setup could capture high-precision strain measurements in a centrally loaded steel plate.

**Table 12:** Recent papers on shearography NDT techniques.

NDT Type	Description	Refs
Dynamic phase-shifting shearography	This paper presented a dynamic phase-shifting shearography system based on a common-path interferometer. The theoretical analysis showed that shearography applications could be more reliable using the proposed system.	[224]
Digital shearography	A single-camera digital shearography system was proposed with dual sensitivity to measure minor and extensive shearograms within the same measurement field. Experiments were conducted to evaluate the system’s performance for dynamic imaging of an object with defects.	[225]
Acoustic shearography	This paper used acoustic shearography imaging to detect defects in carbon fiber composite materials. The results of acoustic shearography provided sufficient defect imaging with significantly reduced imaging time compared to X-ray computed tomography.	[226]
FEM-assisted shearography with spatially modulated heating	Shearography was advanced towards a quantitative inspection tool for thick composites utilizing the finite element method (FEM). According to the results, the proposed spatially modulated heating (SMH) approach improved deep defect detection in thick composites by 2 to 3 times compared to global heating (GH).	[227]
2D shearography	A two-dimensional (2D) shearography with source displacement was proposed for measuring object contours. In the experimental work, spherical and hyperbolic paraboloid surfaces and objects with different types of surfaces were tested, and contours were successfully measured.	[228]
Dual shearing direction shearography	Based on a spatial light modulator (SLM), this study proposed a dual shearing shearography system. Its simple structure, relative light efficiency, and good phase map quality make this system more advantageous than spatial phase shift shearography.	[229]
Spatial phase-shift shearography	Dual-direction sheared spatial phase-shift digital shearography (DDS-SPS-DS) were proposed to measure strain/displacement derivatives simultaneously. Two Michelson Interferometers were used as shearing devices to create two shearograms, one in the x-shearing direction and one in the y-shearing direction. A single CCD camera was used for the recording.	[230]
Pixelated carrier phase-shifting shearography	The aim of this work was to develop a pixelated carrier phase-shifting shearography based on a spatial-temporal low-pass filtering algorithm. By simultaneously low-pass filtering the phase maps in the spatial and temporal domains using the algorithm in the complex domain, the phase maps showed better phase quality.	[231]

#### 3.3.4. Infrared Technologies

**Figure 7:**
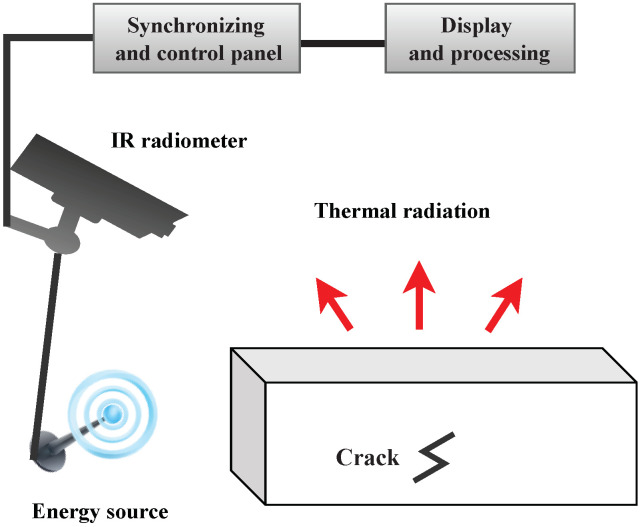
Schematic of the measurement principles of infrared thermography testing.

Infrared technologies were first discovered in the early 19th century. In infrared testing, temperature changes on the surface of an object are monitored over time using thermographic technology [232,233,234,235]. Through an infrared detector, infrared thermography maps thermal patterns on an object’s surface in a non-intrusive and contactless manner. Surface images with pronounced thermal patterns are produced indicating not only the surface but also subsurface irregularities. As is shown in Figure 7, a typical infrared thermography system comprises the following components: an IR radiometer, energy source, control panel, and data processor. A comprehensive review paper on this topic can be found in [236] for readers interested in the topic. In the following, we address the advantages and limitations:

- Advantages: Affordable and fast operation; real-time implementation; compatible with a variety of materials; damage visualization; single-sided inspection; safe procedure (non-ionizing radiation).
- Disadvantages: Delicate equipment, unsuitable for field testing; the precision is affected depending on the specimen geometries and complexities; restrictions are imposed due to the expense and accessibility of excitation sources in the field; computing power and algorithms determine the processing time for data; identifying cracks will require higher automation from footage; offshore structures may have difficulty implementing the application.

Five critical characteristics of infrared technology are addressed in the following:

- Wavelengths and electromagnetic spectrum: Radiation is characterized by its wavelength and frequency. The electromagnetic spectrum covers waves with frequencies ranging from below 1 Hz to above $10^{25}$ Hz. Infrared radiation covers a range from roughly 300 GHz to 400 THz (1 mm–0.78 μm) and is invisible to the human eye. Several sub-divisions exist in the infrared spectrum, with each having its own characteristics:
  – NIR (near infrared): These are wavelengths in the infrared spectrum close to the visible spectrum, between 0.78 μm and 2.5 μm.
  – SWIR (short wave infrared): The spectrum from 1 μm to 2.7 μm.
  – MWIR (medium wave infrared): The spectrum from 3 μm to 5 μm.
  – LWIR (long wave infrared): The spectrum from 7 μm to 14 μm.
  – FIR (far infrared): The spectrum from 14 μm to 1000 μm.
- Detector: An infrared detector can detect radiation from the infrared spectrum. Current detectors can be classified into two types:
  – Cooled: These detectors are maintained at shallow temperatures using cryogenic cooling. This system lowers the sensor temperature to cryogenic temperatures, which reduces heat-induced noise to a level lower than the scene’s signal.
  – Uncooled or microbolometers: These detectors do not need a cooling system. Here, the temperature of the microbolometer changes when temperature differences occur in a scene.
- Indicator of detector sensitivity: Thermal sensitivity is measured by NETD (noise-equivalent temperature difference). This is the smallest temperature difference that a camera is capable of detecting. The temperature is expressed in milliKelvin (mK) or degrees Celsius (°C). NETD determines how well a camera can detect thermal contrast the lower the number, the better the sensitivity. In terms of contrast, NETDs are analogous to visible light detectors.
- Resolution and field of view (FOV): A camera’s FOV indicates the range/angle of light that can be captured. The FOV of an image must be considered in conjunction with its resolution (the number of pixels).
- Analog or digital: Analogue-to-digital converters (ADCs) convert analog signals to digital (binary) signals. Digital-to-analog converters (DACs) convert digital signals into analog signals.

Two different types of infrared technology currently exist [232,237]: (1) infrared technology that detects infrared radiation and (2) technology that transmits information via infrared radiation (called wireless infrared technology).

The first type of infrared technology utilizes sensors that convert infrared radiation into usable information. Thermal imaging is a typical example of infrared technology. Since heat releases infrared radiation, the source of the heat can be identified using infrared radiation detection technology. Thermal imaging cameras can collect data about different temperatures (such as infrared radiation levels) and convert them into heat-mapping images that are invisible to the naked eye. Military and civilian industries use thermal imaging cameras for a variety of purposes. Since infrared light can pass through thick areas of dust and cloud, astronomers have developed tools that detect infrared radiation in space, enabling them to study aspects of the universe that are not visible to the naked eye.

The corresponding infrared sensors are electronic instruments that detect specific characteristics of their surroundings by either emitting or detecting infrared radiation. They are capable of detecting motion and measuring the heat being emitted by objects. Three laws govern the physics behind infrared sensors:

- Planck’s Radiation Law: Radiation is emitted by every object at a temperature T not equal to 0 Kelvin.
- Stephan Boltzmann Law: The total energy emitted by a black body at all wavelengths is proportional to its absolute temperature.
- Wein’s Displacement Law: Different objects emit different wavelength spectra at different temperatures.

The infrared sensors can be active or passive [238] and can generally be divided into thermal and quantum infrared sensors. Thermal infrared sensors use infrared energy as a heat source. They do not require cooling but have slow response times and low detection capabilities. No relationship exists between their photosensitivity and their wavelength capabilities. Quantum infrared sensors, on the other hand, offer higher detection capabilities and faster response times. Since photosensitivity depends on the wavelength, quantum detectors need to be cooled for highly accurate measurements.

Wireless infrared technology uses infrared radiation to transmit data and commands rather than detect them. An example of wireless infrared technology is a TV remote control. A remote’s infrared sensor transmits a signal to a TV’s sensor, which sends a command to the television (for example, turning it on or turning up the volume). Wireless infrared technologies can be classified as directed or diffuse. Infrared lasers transmit information through directed technologies, but the receiver and the source must be unobstructed. Infrared light disruption technology can be used to detect whether a predefined threshold has been crossed. With diffuse infrared technology, the transmitted beam is scattered, making it more difficult to block. The TV remote is an example of diffuse infrared wireless technology: it will work as long as it is used in the same room. Further examples of wireless infrared technology are intrusion detectors, home entertainment control systems, robot control systems, medium-range, line-of-sight laser communications, cordless microphones, headsets, modems, and printers.

Three main methods of active infrared thermography NDT exist: optical infrared thermography [239], ultrasonic infrared thermography [240], and microwave thermography [241]. Heinz et al. [242] examined infrared thermography (IRT) for analyzing steel rope failures. A thermal imaging camera recorded the relationship between temperature increase and force increase. The research found that when the temperature rises, the steel rope’s minimum load capacity decreases by 43.01%, as monitored by a thermo-camera. The thermo-camera’s mounting affected the accuracy of the measurements. In [243], Deane et al. aimed to examine the effectiveness and challenges of NDT using active IRT to inspect aerospace-grade composite samples, using the signal-to-noise ratio (SNR) as a performance parameter to compare uncooled and cooled thermal cameras. Both types of cameras were compared using seven different SNR definitions to determine whether a lower-resolution uncooled IR camera could meet NDT standards. Using active IR thermography to evaluate coating thickness is a common technique for NDT of materials. To demonstrate the feasibility of this method, Moskovchenko et al. [151] described a one-sided thermal NDT procedure that employed apparent effusivity as a quantitative method for evaluating coating thickness. The proposed algorithm determined a threshold value of apparent effusivity based on specific coating-on-substrate structures. Swiderski [244] investigated the possibility of using IR thermography methods to detect defects in ballistic covers made from carbon fiber-reinforced composites used in military vehicles. An overview of IRT fault diagnosis for renewable and sustainable energy (RSE) systems is presented in [245].

Recently proposed matched filter-based non-periodic infrared thermographic approaches have become increasingly popular for various NDT methods, including pulse-based and mono-frequency excited modulated lock-in thermography. These approaches lead to superior test resolution and sensitivity for detecting hidden defects in the test material over pulse-based and mono-frequency excited modulated lock-in thermography. As a result, pulse compression favorable techniques are more economical and reliable than conventional pulse-based thermographic techniques because they can be implemented in moderate experimentation times compared to mono-frequency lock-in thermographic techniques. Dua et al. [246] demonstrated the advantages of pulse compression favorable frequency-modulated thermal wave imaging for identifying flat bottom holes in polymers. To demonstrate how IRT and ANN can be combined, Chulkov et al. [247] trained and verified a neural network to determine defect depth in infrared thermographic NDT using ten different sets of input data. The input data sets included raw temperature data, polynomial fitting, principal component analysis, Fourier transforms, and other parameters. With polynomial fitting in logarithmic coordinates and further computation of the first temperature derivatives, a minimum error of 0.02 mm for defects in CFRP at depths from 0.5 to 2.5 mm was achieved. One of the novel IRT techniques is atomic force microscopy-based infrared spectroscopy (AFM-IR) [248]. AFM-IR detects localized thermal expansion in a sample caused by the absorption of infrared radiation using the tip of an AFM probe. The combination of AFM-IR and infrared spectroscopy can thus provide the spatial resolution of AFM along with chemical analysis and compositional imaging capabilities.

#### 3.3.5. Radiographic Technologies

Radiographic testing (RT) enables the internal inspection of an object. RT penetrates materials by applying short wavelength electromagnetic radiation, such as X-rays or Gamma rays [249]. As waves penetrate and pass through a material, the variations of radiation detected on the opposite side can be measured to determine its thickness or composition. Several sources of radioactive material produce high-energy photons, including Ir-192, Co-60, and Cs-137 [250]. As an extension of radiographic testing, neutron imaging uses neutrons instead of photons to penetrate materials. In neutron radiography, images are generated by analyzing neutron attenuation. Although there are similarities between neutron radiography and X-ray imaging techniques, in neutron radiography, some material artifacts that would otherwise be visible through X-ray imaging may stay hidden. For example, neutrons can easily pass through lead and steel; however, they cannot pass through plastics, water, and oils. Radiographic testing is widespread in the aerospace, defense, off-shore, marine, energy, petrochemical, waste management, automotive, manufacturing, and transportation industries.

The following summarizes the advantages and limitations of radiographic testing:

- Advantages: Detection of surface and internal flaws; ability to inspect hidden areas; minimal preparation; few material limitations; high resolution
- Disadvantages: Health and environmental impact of radiation; requirement of safety protective equipment; high degree of skills and experience; slow process; only qualitative but not quantitative results; high voltage requirements; expensive; ineffective for planar and surface defects.

RT is used in mining, petrochemical, steel fabrication, and oil and gas industries to inspect the internal structure of parts, welds, or materials for discontinuities. In the field of NDT and SHM systems, RT is used for:

- Analyzing variations in structures;
- Analyzing material thicknesses and tracking changes;
- Detection of cracks and voids;
- Tracking minute surface changes;
- Assessing porosity.

In RT, there are four leading RT technologies: film, computed radiography, fluoroscopy, and digital detector arrays, each of which has advantages and disadvantages.

- Film radiography: Although this technology has been around for over 100 years, it is still highly regarded and widely used. Film radiography has a high spatial resolution and provides high-quality results. However, the film is produced with potentially harmful chemicals, and the development is time intensive.
- Computed radiography (CR): In CR, a phosphor imaging plate is used instead of a film. This requires less exposure time, and results are obtained quickly and without chemical processing. In addition, phosphor plates have an excellent dynamic range and are more sensitive than films. The image captured on a phosphor plate is converted into a digital signal which can be viewed on a computer monitor and stored digitally for later review and archive.
- Fluoroscopy: Fluoroscopy enables continuous and real-time examination of large structures, making it a cost-effective and practical solution. As a result, this method is not as sensitive as others and provides images of lower resolution.
- Digital detector array (DDA) radiography: Furthermore, this method, known as direct radiography, is similar to computed radiography. In digital imaging, an electronic flat panel detector captures and displays an image directly on a computer screen instead of using a film scanner. It produces higher-quality images and has shorter exposure times than CR, but it is more expensive and less portable than CR or film.

In [251], Li et al. discussed the operation of digital radiography (DR) testing and how to configure instrument parameters. The researchers found that digital ray testing was an effective NDT technology for quality testing of the online insulation pipelines. A novel power line inspection robot based on DR was designed and applied by Gao et al. [109] for the NDT of overhead aluminum conductor composite core (ACCC) wires. Potential defects were detected using a deep-learning-based defect diagnosis method in conjunction with manual diagnosis. In conventional radiography, images are often impeded by the superimposition of the object’s structure and scattering of X-rays. Kim et al. [252] presented a new method of restoring images based on a simple radiographic scattering model in which the direct transmission function and the intensity of scattered X-rays were estimated from a single X-ray image. Yenumula et al. [253] presented case studies showing how DR imaging modalities can assess structural integrity and analyze failures in industrial components. Chen and Juang [254] used X-ray image detection based on radiography methods combined with deep learning methods for the NDE of aircraft. Boaretto and Centeno [255] presented a method for detecting and classifying defects in radiographic images of welded joints using the double wall double imaging (DWDI) exposure technique. Kolkoori et al. [256] proposed a new non-destructive imaging technique for aerospace materials using X-ray backscatter (XBT). An edge detection model with improved performance was presented by Xiao et al. [257] using a convolutional neural network (CNN) and a Laplacian filter. The proposed CNN model successfully detected fuzzy defects in noisy X-ray images by constructing datasets with varying noise levels. Compared to conventional edge detection algorithms, Canny and SUSAN, the detected information had a better structure similarity. A brief overview of industrial radiographic imaging techniques for verifying industrial specimen stability can be found in [258]. Furthermore, Udod et al. [259] presented a review of the modern state and practical applications of various forms of digital radiography. Further information on the latest RT in NDT systems can be found in [260,261,262].

### 3.4. Ground Penetrating Radar

**Figure 8:**
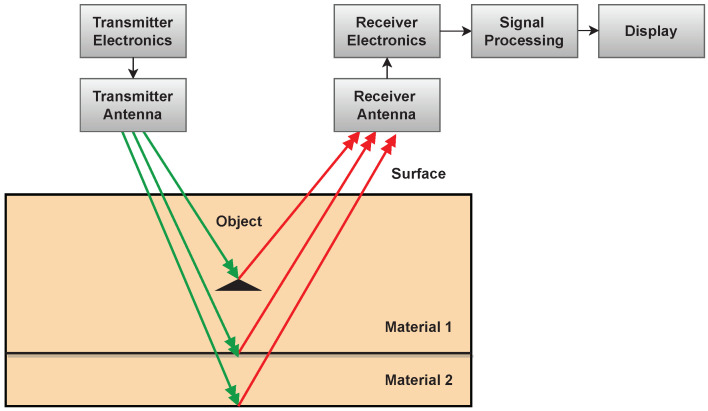
Scheme of GPR technology.

Ground-penetrating radar (GPR) is a geophysical method that uses radar pulses to image subsurface features [263]. The method was initially developed for the mapping of soil but was adopted as an NDT method for the assessment of various structures and materials, including concrete, asphalt, metals, pipes, cables, or masonry. This nondestructive method utilizes electromagnetic radiation in the radio spectrum’s microwave band (UHF/VHF frequencies), typically between 10 MHz and 2.6 GHz, to detect reflected signals from subsurface structures. Electromagnetic energy is emitted into the media by a GPR transmitter and antenna. When the energy encounters a change in material, such as damage, or a boundary between materials of different permittivities, such as buried objects, it may be reflected, refracted, or scattered back to the surface. GPR uses the same principles as seismology, except that instead of acoustic energy, GPR uses electromagnetic energy. Here, the energy may be reflected at boundaries whose electrical properties change rather than their mechanical properties, as with seismic energy. As illustrated in Figure 8, radiation is emitted by a transmitting antenna and reflected from material changes and embedded objects. A receiving antenna records the reflected waves, and the data are transmitted for further signal processing.

The effective depth range of GPR may be limited by the material’s electrical conductivity, the transmitted center frequency, and the radiated power [264]. An increase in electrical conductivity causes attenuation of electromagnetic waves; therefore, the penetration depth decreases with increased electrical conductivity. Generally, higher frequencies have a shallower penetration depth than lower frequencies due to frequency-dependent attenuation mechanisms. Higher frequencies, however, result in a higher resolution. Therefore, the choice of operating frequency is always a trade-off between resolution and penetration. The optimal depth of subsurface penetration is achieved in ice, where penetration depth can reach several thousand meters (e.g., to the bedrock in Greenland) at low GPR frequencies. In dry sandy soils or solid dry materials like granite, limestone, and concrete, the penetration depth could reach 15 meters. For materials with high electrical conductivity and moist or clay-laden soils, penetration may be as little as a few centimeters. GPR antennas are generally in contact with the test object to achieve the most robust signal strength; however, GPR air-launched antennas can be used above the object’s surface.

In the following, the advantages and limitations of GPR testing are addressed:

- Advantages: Non-invasive, non-destructive, and safe to use in public areas; fast data collection; suitable for large-area scanning; accurate depth, location, and dimension assessment of small and large objects; scanning at different depths and resolutions is possible with multiple frequencies; interpreting data in real-time or processing it off-site is possible; subsurface scanning is faster, safer, and more affordable than other methods.
- Disadvantages: Signal scattering limits performance in heterogeneous conditions (e.g., rocky soils); interpretation of radargrams is generally non-intuitive to the novice; designing, conducting, and interpreting GPR surveys requires considerable expertise; for extensive field surveys, relatively high energy consumption can be problematic.

The transmitter and receiver designs for GPR systems generally fall into two categories [265]. The first is based on using short EM impulses and is known as an impulse radar system. As a second type of radar, step-frequency or continuous-wave radars sweep sinusoidal waves over a range of frequencies. An impulse radar is the most common type of GPR system in commercial use.

Soil classification is one application of GPR. Conventional methods for classifying soil types are time-consuming, invasive, and expensive. GPR is highly suitable for classifying soil types and provides a fast, non-invasive, cost-effective alternative. For civil engineering structures, GPR is used for several applications, including the assessment of bridges, tunnels, pavements, and buildings, as well as for underground utilities and voids. The COST Action TU1208 addressed various aspects of GPR for civil applications [266]. A review of GPR methods for investigating reinforced concrete structures was presented by Tosti and Ferrante [267]. A GPR test was conducted by Morris et al. [268] on the Streicker Bridge on Princeton University’s campus with embedded fiber-optic strain and temperature sensors to investigate the effectiveness of attribute analysis techniques. According to [269], experimental activities were conducted inside a camp in Shanghai, China. GPR was used to detect and locate rebars in columns, beams, and floors to assess the concrete structures of the building.

An NDT technique for pavement distress detection based on GPR and network in networks has been proposed by Tong et al. [270]. As input data, GPR signals were directly imported into two network-in-network structures. Garcia-Fernandez et al. [271] developed a novel airborne subsurface sensing and imaging system consisting of a GPR mounted on an unmanned aerial vehicle (UAV). Santos-Assunçao et al. [272] used GPR combined with seismic tomography on one column to obtain the maximum amount of information on the structure. A review of the latest GPR developments for infrastructure applications was presented by Lai et al. [273] and included the assessment of bridges, pavements, tunnel liners, buildings, and geotechnical and buried utility systems. Sossa et al. [274] conducted several laboratory tests to investigate how different corrosion stages affected GPR signals. Using GPR data analysis and different estimation approaches based on material dielectric properties, Loizos and Plati [275] evaluated the accuracy of estimating asphalt layer thickness. Giannopoulos [276] presented a software tool for modeling GPR responses from arbitrarily complex targets. In recent years, deep learning algorithms have been investigated for their effectiveness in extracting features from GPR data. Barkataki et al. [277] proposed a deep convolutional neural network (CNN) model for automatically identifying soil types using data from GPR testing. Tong et al. [278] examined methods involving deep learning and GPR to inspect civil engineering structures and classified them based on the data types they used.

### 3.5. Laser Scanning Photogrammetry

Laser scanning measures distances by steering/directing laser beams in a line or within an area (2D or 3D). The operation of a laser scanner can be either stationary or mobile. Laser scanners are typically mounted on a tripod when used in stationary mode, but they can also be mounted on a vehicle, robot, or aircraft when used in mobile mode. Digital photogrammetry and laser scanning are combined in laser scanning photogrammetry. By applying image processing, digital photogrammetry can generate digital terrain models (DTMs), digital surface models (DSMs), and orthoimages. Objects can be reconstructed and classified for mapping and visualization. A comparison of laser scanning and digital photogrammetry is presented in Table 13 [279]. In laser scanning photogrammetry, six key components are necessary: (i) a laser scanner, (ii) a digital camera, (iii) a global positioning system (GPS) (when the laser scanner’s position is changed), (iv) a total station (optional), (v) a portable computer, and (vi) a data processing software.

In the following, the advantages and limitations of laser scanning photogrammetry are addressed:

- Advantages: Rapid and thorough technology; high accuracy; non-contact testing; cost-effective; safe.
- Disadvantages: Accuracy reduction from light interferences of ambient sources; high initial investment; incapable of measuring surfaces beyond the range of the scanner.

Napolitano et al. [280] developed a laser scanning technique for assessing plastered masonry walls in Palazzo Vecchio. The wall geometry was captured using laser scanners, and the crack severity in the plaster layer was documented by terrestrial photogrammetry and high-resolution images. In unit-based masonry, Laefer et al. [281] formulated basic mathematics to determine the minimum crack width detectable by a terrestrial laser scanner. Through the combination of a laser range finder (Leica Zinder Distro Plus) and a digital camera, Rodríguez et al. [282] developed a portable laser photogrammetric sensor. As part of a photogrammetric rock cut survey, Sturzenegger and Stead [283] employed 3-D laser scanning photogrammetry, a digital camera, and data processing software to perform laser scanning photogrammetry. In [284], 3D laser scanning technology was used to detect surface potholes on asphalt pavements. Wang et al. compiled a comprehensive overview of laser scanning photogrammetry technologies in [279].

### 3.6. Other NDT Approaches

In addition to the NDT techniques discussed above, several other techniques have been developed that are less established. As the demand for NDT grows, developing new technologies is an ever-growing area of research. Some novel techniques are limited to only a few or even one peer-reviewed scientific paper due to the novelty of the concepts. Several characteristics of these techniques make them attractive, but limitations also hinder their application. In addition to developing advanced disruptive technologies, existing methods have also evolved considerably. As an example, new developments of laser-based methods include laser digital shearography, laser holographic interferometry, laser interferometry, laser interferometry or electronic speckle pattern interferometry, laser Doppler vibrometry, laser ultrasound, laser diffraction grating, laser acoustic-emission (laser-AE), laser infrared photothermal radiometry (laser IR-PTR), laser photoacoustic spectroscopy, and laser wireless power transmission [279].

Other NDT strategies include the following:

- Dynamometer testing: This technique was developed to identify potential slipping in the high-speed shaft tapered roller bearings in drive train systems [285];
- Sound-based monitoring using audio speakers: This method was used to ensonify the internal cavities of WT blades and arrays of external microphones detecting patterns in airborne sound waves [286];
- Short-range Doppler radar: This technique was recently tested for on-site SHM of wind turbine blades [287];
- Multi-sensor apparatuses: This approach uses multiple types of sensors, such as optical, acoustic, and vibrational sensors, to detect bird strikes and bad strikes [288].

Despite significant advances in NDT, current techniques encounter many challenges; the most pressing is analyzing and interpreting the large amount of data collected during testing, which makes the techniques very time-consuming and only operable with highly skilled personnel. Artificial intelligence and machine learning are possible solutions to these challenges in data processing and pattern recognition [289]. In addition, robotics, artificial algorithms, and network coding can minimize human errors by automatically inspecting and identifying flaws and defects.

For decades, scientists and engineers have developed signal processing and statistical analysis approaches to resolve problems in NDT, especially the interpretation of NDT signals for flaw detection and characterization. For example, clustering (the identification of natural clusters in collected signals) has been demonstrated to help analyze acoustic emission signals successfully [290]. Different types of acoustic emission signals can be separated using clusters in signals. A correlation exists between these signals and defects, such as fiber breakage, matrix cracking, and interface failure [291]. Matrix decomposition techniques have been used in combination with machine learning for guided wave prognostic and health management (PHM). This approach can help separate damage events and identify changes over an extended period of guided wave measurements, such as 10 to 1000 to 100,000 s. Further, it can be used to obtain information from datasets in the presence of noise, and temperature variation [292]. In NDT applications, neural networks have been used to classify ultrasonic signals for crack detection [293]. This method can locate defects, characterize material properties, and analyze the damage. NDT’s future is currently heading towards data-driven approaches involving deep learning, transfer learning, and physics-based machine learning. Multiple articles have been published in this domain [294].

Among the future directions of NDT are studies of new sensing modalities, such as frequency-modulated continuous wave (FMCW) radar sensing for wind turbine blades, as proposed by Tang et al. [295]. Various material characterization challenges have been addressed using this technology, including identifying damage for surface and subsurface analysis using static and dynamic loads [296]. This sensing mechanism demonstrated its potential for detecting and preventing further blade degradation, thereby increasing wind turbine blade efficiency. Using FMCW radar sensing for real-time defect detection is an advanced approach suitable for SHM. Due to its non-contact and non-destructive nature, FMCW radar sensors are unaffected by smoke, mist, and fog. FMCW systems can significantly enhance the current state-of-the-art defect detection approaches, such as visual and thermal inspections, by coupling novel digital analysis and digital twin systems [297].

Using data-driven analysis in sensing technologies has become an advanced and rapidly evolving technique. Due to the increasing complexity of systems and dependencies across system networks, such as NDE techniques, digital tools are becoming increasingly popular in integrating information and data from distributed monitoring systems. Digital twins (DTs) are digital representations of devices and physical systems, similar to mirrors of physical objects that link physical and virtual objects. Human-object interactions can be improved directly and spontaneously with less specialist knowledge required from end users. Evaluations of the DT framework are currently ongoing in real-world scenarios, such as robotic platforms, to demonstrate that operators can fully interact with their physical assets via the internet and a simulated virtual workspace [298]. This implementation of advanced technologies will provide NDT methods with a great deal of potential.

## 4. SHM Systems

SHM started attracting attention in the research community in the 1980s and was first applied in offshore platforms and aerospace structures. Over the following decades, SHM gained further popularity in research as well as asset management, and SHM systems have been implemented to monitor various types of structures, such as bridges, wind turbines, buildings, pipelines, or railway tracks [299]. Several authors have described the concept of SHM in terms of its aims. Here are a few examples:

- In SHM, existing civil structures are characterized to identify and detect structural defects [300].
- SHM involves continuously interrogating sensors installed within a structure to extract characteristics indicative of the structure’s current state [301].
- In SHM, different parts of the structure are continuously diagnosed and assembled as a whole so that the entire structure can be diagnosed continuously [302].

**Figure 9:**
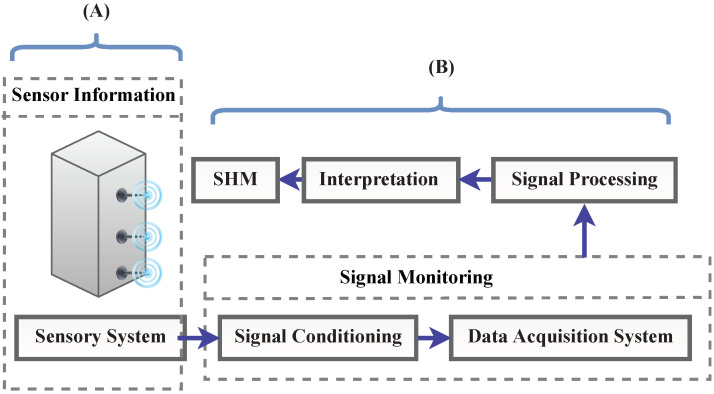
Components of a general SHM system—(**A**) sensing and (**B**) data analysis.

SHM systems should meet the following requirements: being affordable; able to continuously assess a system; capable of adapting to environmental changes; able to detect diverse types of damages; robust to measurement noise and ambient loads. In general, SHM involves two major processes (a) sensing and (b) data analysis. Typical components of an SHM system are the sensory system (active or passive), data acquisition and signal conditioning, data transfer and storage mechanisms, data management, signal processing, and data interpretation. Figure 9 illustrates the general schematic of an SHM system.

Several bridges in the world have been equipped with SHM systems using different types of sensors and data analysis techniques. Table 14 presents a selection of bridges currently being monitored using SHM systems.

### 4.1. Benefits of SHM Technologies

The implementation of SHM technologies can have many benefits and improve several aspects of civil structure management and design, including the following:

- Active safety monitoring and control: Alert systems can inform asset managers if prescribed limits are exceeded, such as when abnormalities in load or responses occur, ensuring human and structure safety.
- Environmental monitoring: Site-specific environmental conditions such as wind and temperature can be evaluated.
- Improved structure assessment: The reliability and accuracy of structural assessments can be improved based on up-to-date structural response data.
- Optimization of maintenance: An optimal inspection, maintenance, and repair schedule can be determined on an “as needed” basis when indicated by monitoring data, resulting in cost reductions.
- Real-time safety assessments can be performed during normal operations or immediately following extreme events.
- Assumptions and parameters related to the structure design can be validated, resulting in improvements in future specifications and guidelines.
- Future performance can be predicted based on past and current monitoring data.

Human safety is the most obvious advantage. Disasters such as bridge collapses have motivated much research on SHM strategies. If employed as an early warning system related to safety problems, SHM strategies can be highly beneficial, even at a minimum level, e.g., detecting damage or strength degradation. Furthermore, an automated SHM system can assess the safety of inaccessible areas, which may otherwise remain hidden from visual inspection. Implementing sophisticated SHM systems may also lead to other benefits, such as policy changes. Currently, routine inspections and maintenance of civil structures are implemented at specific intervals following standard procedures. This time-based approach implies that unexpected failures between scheduled inspections may be overlooked, leading to life-threatening situations. On the other hand, civil structures may be subjected to unreasonably conservative inspection schedules resulting in unnecessary costs. The economic impact can be even more significant if structural components are replaced as part of routine maintenance, where even healthy components are renewed. Since SHM is aimed at continuous monitoring, and structural maintenance is condition-based, SHM strategies may solve both sides of this problem. In addition to reducing downtime for routine maintenance, condition-based maintenance schedules can reduce emergency maintenance downtime. Consequently, this would benefit safety, structure operation, the economy, and the environment [11].

### 4.2. Environmental and Operational Conditions (EOCs) Effects

**Figure 10:**
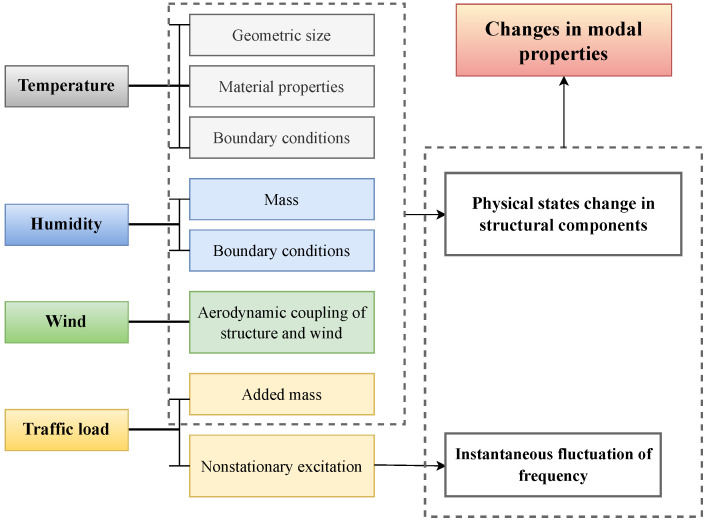
Influence mechanisms of EOCs on modal properties.

Civil engineering structures are typically subjected to changing EOCs, which can impact the structural parameters and responses. Since SHM systems capture time-variant data, these EOCs can heavily affect measurement signals. Hence, a significant challenge of SHM systems is their sensitivity to EOCs parameters. Various SHM techniques have been proposed to determine the extent and location of damage in in-service structures considering the effects of EOCs variations [330]. Environmental factors include temperature, humidity, wind, seismic actions, settlement, and scouring. Operational factors include highway, traffic, railway, ship impact, and permanent loads. Changing EOCs may have more significant impacts on structures than damage-induced changes. Neglecting these influences may affect the accuracy of damage detection and lead to incorrect conclusions. Thus, sensors that are robust to EOCs variations can be highly advantageous in reliably and accurately detecting damage in structures. Table 15 lists typical EOCs affecting civil infrastructure and sensors suitable for measuring EOCs in SHM systems. An important aspect of damage identification methods is the selection of attributes that discriminate between damaged and healthy structures. In SHM, these features are commonly constructed from the dynamic properties of structures (e.g., modal properties), known as vibration-based approaches. However, a structure’s modal properties are sensitive to both damage and EOCs variations. Any change in EOCs alters the structural stiffness and mass, which affect the modal properties and hence can be mistaken for damage. Figure 10 illustrates the influence mechanisms of various EOCs on modal properties.

In SHM, the effects of temperature on structural modal variations have been extensively evaluated and are considered one of the dominant interfering factors. The influence mechanisms of temperature variation are mainly (i) changes to the geometrical dimensions due to thermal expansion and contraction (these deformations are, however, relatively negligible); (ii) changes to the mechanical properties, such as the elastic modulus, which is the primary reason for structural stiffness changes; and (iii) changes in boundary conditions and internal forces in a statically indeterminate system [331]. Peeters et al. [332] reported that the first four modal frequencies of the Z24 bridge varied by 14–18% due to a decrease in the temperature of the bridge deck and support to 0 °C and below.

The wind load is characterized by two main features—speed and direction—influencing the modal parameters, response amplitude, and aerodynamic coupling between the structure and wind. Especially for bridge structures, the modal frequencies fluctuate due to wind excitation. Wind-induced vibration has a more significant energy input than damping when a system is subjected to strong winds or typhoons, causing it to flutter or buffet. According to [333,334], the modal frequencies decrease with an increase in wind speed. For low wind speeds, however, a poor correlation exists between frequencies and wind speed.

Variations caused by humidity include changes in structural mass and boundary conditions. These changes occur slowly as the moisture content increases and tend to reduce the structural frequencies. According to [335], humidity has only a small effect on the stiffness in long-span structures.

Structural changes due to traffic loading primarily result from added mass and nonstationary excitations acting on the structure. These changes typically undergo daily or weekly fluctuation patterns. As mass increases, the modal frequency decreases, as can be interpreted from an equivalent spring-mass model of traffic flow or as the root-mean-square of acceleration. Nonstationary excitation mainly changes the stiffness of a system with vibration amplitude changes causing random fluctuations [336].

Using data-driven normalization methods, Cavadas et al. [337] demonstrated that EOCs variations on modal properties can be predicted by applying multivariate statistical analysis techniques. The analysis data should cover at least one year to characterize the influences of EOC variations on the structural modal properties. Both engineering and academic fields have given significant attention to data-driven normalization methods in the context of EOCs and SHM. These methods can be divided into two basic categories:

- Input–output methods include multiple polynomial regression (MPR), multiple linear regression (MLR), and support vector regression (SVR).
- Output–only methods include principal component analysis (PCA), cointegration analysis (CA), auto-associative neural network (AANN), and Mahalanobis squared distance (MSD).

### 4.3. SHM Strategies

**Figure 11:**
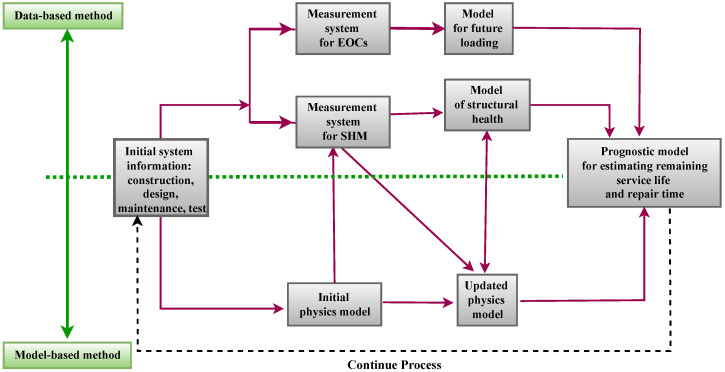
Flowchart of model-based and data-based methods.

Individual objectives, aims, and constraints of asset management have led to the development of different strategies and approaches for SHM. There are two types of SHM strategies model-based and data-based methods [338]. Figure 11 presents a flowchart providing a general overview of model-based and data-based SHM. According to the figure, damage detection processes in model-based methods are based on initial physics models of structures. Damage can be determined by updating the initial properties of structures and comparing them with real properties. Data-based methods are based on measurements. The effects of EOC variations can also be considered using these methods. In Table 16, some recent model-based and data-based studies are presented.

#### 4.3.1. Model-Based SHM Systems

In model-based SHM, a structural model is employed to identify and assess structural damage and to indicate structural responses to future potential loading conditions and system configurations. The structural model is a type of physical model, e.g., in the form of a finite element (FE) model, and is usually constructed by analyzing design and testing results [339]. The structural models are designed and calibrated using measurements from the actual structure. The underlying physics of a structural system is explicitly represented in structural models, as opposed to data-based techniques that directly interpret measurement data.

Structural models incorporate kinematics, continuity, equilibrium, boundary conditions, and force–displacement relationships. Employing these models makes it possible to simulate structural behavior for a wide range of critical loading conditions. Thereby, the models can diagnose the causes of behavioral changes and determine the impact of such changes on the system’s overall performance. FE models are the most common structural models used to identify damage in engineering structures. Since FE models are simplified representations of the actual structure (materials, geometry, boundary conditions, etc.), model predictions may be erroneous and differ from the actual behavior of structures [344]. With the availability of continuous monitoring data, an FE model can be continuously updated to provide a time-based evolution of the structural model. Consequently, model evolution and probabilistic analysis can be used to predict the structure’s performance in the future and its remaining service life. A modal model is another model commonly used to identify structural damage. These models include modal parameters, such as modal frequencies, mode shapes, and modal damping ratios. Modal models differ from structural models in that the modal model lacks specific information on the structural connectivity, stiffness distributions, and damping distributions of a structure. It may be more convenient to express structural behavior through modal models since modal parameters describe the resonant spatial and material behavior [343].

In the following, we address the advantages and disadvantages of model-based SHM systems:

- Advantages: (1) Predictions can be made about the impact of variations in loading and usage, (2) potential consequences of future damage can be evaluated, (3) insights and recommendations for further inspections and measurements can be made, (4) if causal links can be established between measurements and possible causes, data interpretation becomes simpler, (5) rehabilitation and repair planning can be supported, (6) can assist in making better maintenance decisions.
- Disadvantages: (1) Modeling is time-consuming and costly, (2) modeling errors may result in false identifications and predictions, (3) managing a large number of candidate models can be difficult, (4) several interpretation-measurement cycles may be needed to identify the correct model, and (5) combinatorial challenges may arise with complex structures.

#### 4.3.2. Data-Based SHM Systems

In data-based SHM, the current condition of a system is assessed using measurements from previously recorded data. The primary objective of data-based SHM strategies is the detection of anomalies in structural behavior, and pattern recognition is its underlying principle. Data-based techniques can detect changes in system configurations or load conditions, including structural damage. Abnormalities are detected due to a difference between the recorded measurements over the previous period. There is no need to develop a model of the system behavior, and the simplicity of implementation makes data-based SHM suitable for various structures. Data-based methodologies are entirely data-driven and do not provide any information regarding the physical processes underlying the data evolution [345]. Further, they operate inadequately when attempting to determine the character of a change, such as damage progression [346]. A large variety of data-driven models have been developed and employed in SHM applications and include methods such as autoregressive and rational polynomial models. These models generally incorporate several strategies, such as data reduction and representation, feature extraction, and abnormal detection.

The following advantages and disadvantages of this method are summarized:

- Advantages: (1) No system model required, (2) a wide range of signal analysis options, (3) no damage studies required, (4) damage accumulation can be tracked by incremental training, and (5) capable of detecting situations requiring model-based interpretation over long periods.
- Disadvantages: (1) Potential misinterpretation of signals, (2) indirect guidance for structural management activities, (3) insufficient capabilities for rehabilitation decisions, and (4) unsuitable for justifying replacement avoidance.

### 4.4. Sensing Systems for SHM

For the design of a suitable sensing system, essential requirements need to be identified. A typical SHM system comprises a network of sensors that measure different structural quantities. The sensor measurements reflect either the structural behavior or external factors, such as environmental or operational conditions, that can influence the sensor readings or behavior of the system. The measured sensor data should be sensitive to damage to allow direct correlations to the structure’s health state and be used for system identification. Examples of conventional sensors used for SHM systems are strain gauges, temperature gauges, accelerometers, and fiber optic-based sensors [347].

The following sensing system characteristics need to be taken into account to design a suitable sensing network:

- Monitoring objectives;
- Sensor types, numbers, and placements;
- Sensor measurement characteristics;
- Sensitivity, bandwidth, and dynamic range;
- Continuous or periodic sampling intervals;
- System installation constraints;
- Power demands;
- Data transmission;
- Telemetry, data acquisition, and storage system;
- Excitation source (for active sensing);
- Memory and processor requirements;
- Resilience of the system in the case of malfunctions;
- Data Analytics.

To select sensors that adhere to the system requirements and restraints, specific sensor characteristics need to be considered, including (1) sensitivity to damage, (2) sensitivity to noise, (3) sensitivity to EOCs variations, (4) sensitivity to chemical influences (5) sensitivity to mechanical influences, (6) measurement accuracy, (7) error-proneness and (8) cost. Most SHM systems consist of a network of multiple sensors, constituting either homogeneous or heterogeneous sensors. A sensor network presents several advantages, such as:

- Increasing the robustness of the intended measurements;
- Enhancing the system’s robustness and reliability;
- Reducing uncertainty in the monitoring results in systems.

A more significant number of low-precision measurements is typically preferred to a smaller number of high-resolution measurements. However, dealing with measurements from many sensors implicates several disadvantages, including the following: (1) The management and analysis of the large volume of recorded data is challenging, (2) the extended sensor network is more prone to environmental effects, and (3) the large volume of data exchange increases the power consumption in the sensor network. Hence, there is always a trade-off between the number of network sensors and the amount of information gathered for an application.

#### 4.4.1. Sensor Technologies for SHM

A critical issue for designing an SHM system is determining the types of sensors suitable for meeting the objectives and scope of the sensing system. Factors to be considered include the type of structure, construction materials, environmental conditions, and possible damage and degradation phenomena. For SHM systems, sensors are primarily used to measure the physical properties of a structure. Sensors suitable for physical measurements can be classified into three categories: kinematics (displacement, velocity, and acceleration measurements), mechanical properties (forces, deformations, stress measurements), and ambient properties (wind and temperature measurements). In Table 17, we present different types of sensors used to measure various physical properties in SHM systems.

Besides classifying sensors by measurement type, they can also be categorized based on various features:

- Active or passive sensing [299]: Active sensors need an excitation or power signal from an external source. On the other hand, passive sensors, do not require any external power source and generate output directly.
- Detection substance [348]: Different substances such as electric, biological, chemical, and radioactive can be detected.
- Conversion method [349]: Conversion types include photoelectricity, thermoelectricity, electrochemistry, electromagnetics, and thermoplastics.
- Analog and digital [349]: Analog sensors measure physical quantities, including voltage or resistance, while digital sensors produce discrete output values (0 and 1’s).
- Application type: For instance, Sehrawat and Gill [350] categorized sensors suitable to the Internet of Things (IoT), including proximity, temperature, humidity, chemical, position, motion, and pressure sensors.
- Other classifications include sensor specifications, material type used, cost, and power source.

The types of sensors used for SHM systems evolved in recent decades, with stain gauges being one of the first sensors to be used. Currently, the most common types of sensors used for SHM include displacement, velocity, acceleration, strain, force, temperature, and pressure sensors [351]. Table 18, Table 19, Table 20, Table 21, Table 22, Table 23 and Table 24 provide a summary of these sensors specifying various sensor types, applications, and their advantages and disadvantages.

Starting in the 1970s, new classes of sensors emerged, fueling the interest in SHM. Newly developed sensing technologies offered new measurement modes, lower cost, and more convenient data acquisition methods. These advanced sensor techniques include fiber optic, wireless, piezoelectric surface, microelectromechanical (MEMS), air-coupled, vision, wireless rechargeable sensor networks, and radar sensor networks. The development of MEMS sensors, for example, allowed sensor packages to be miniaturized while reducing the cost of each sensor. Health-based data interrogation methods were also adopted to infer structural conditions from measurement data. An overview of different types of conventional and advanced sensors suitable for SHM systems is presented in the sections below.

#### 4.4.2. Fiber Optic Sensors (FOSs)

**Figure 12:**
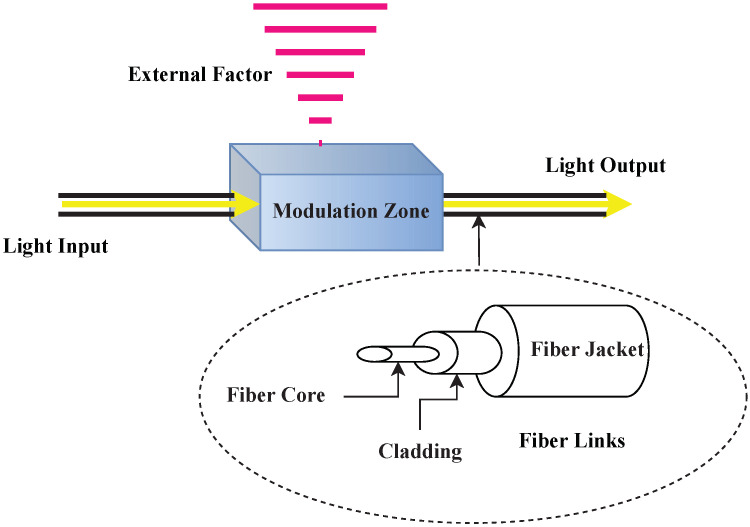
Composition and working principle of a typical FOS system.

Fiber optic sensors consist of optical fibers that are connected to a light source. They use the physical properties of light traveling along a fiber to detect changes in quantities such as strain, temperature, and acceleration. For SHM in civil structures, they are used for various applications, including crack detection, measuring strains, pH levels, vibration, corrosion, and temperature measurements [352]. FOSs analyze the transmitted or reflected light response from an object by analyzing the phase, polarization, intensity, and spectral content of the phase. The optical fiber is a cylinder-shaped, symmetrical structure having a central core with a diameter between 4 and 600 μm. A low refractive index cladding typically surrounds the FOS. Due to the reflection at the interface between the core and cladding, the light waves propagating inside the core are trapped by the cladding. An external plastic coating protects the FOS from the environment and mechanical forces. Figure 12 illustrates the working principle and composition of a typical FOS. In general, FOSs can be classified as point, integrated, quasi-distributed, and distributed sensors based on the spatial distribution of the measurements.

There are several advantages to FOS technology compared to other forms of sensors:

- FOSs have low signal transmission loss enabling remote monitoring and transmission over long distances.
- They are insensitive to electromagnetic interference.
- They offer a great variety of measurable parameters.
- A single optical fiber can have distributed or multiplexed topologies, allowing it to capture full distribution measurements.
- They are corrosion-resistant and have excellent long-term stability and continuous monitoring capability.
- Sensors and cabling are small and lightweight.
- FOS can be permanently integrated into structures.
- They can withstand severe environments such as extreme temperatures, radiation, and vacuum.

As stated above, the main parameters of light that are modulated using fiber optic technology are phase, polarization state, intensity, and wavelength. Sensors can therefore be classified into four categories based on their modulated optical parameter: interferometric sensors, polarimetric sensors, intensity-modulated sensors, and spectrometric sensors. FOSs commonly used to monitor civil infrastructure include Surveillance d’Ouvrages par Fibres Optiques (SOFO) interferometric, Fabry–Pérot interferometric, fiber Bragg gratings (FBG), and distributed Brillouin and Raman scattering sensors.

**Figure 13:**
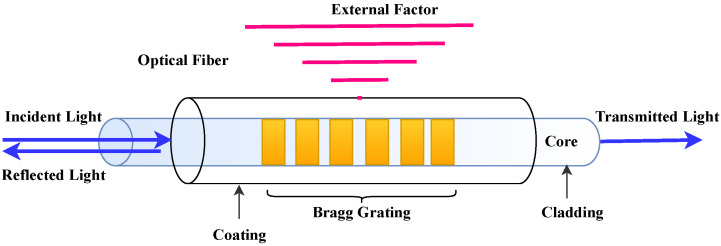
Structure of FBG sensor.

- SOFO sensors [353]: Surveillance d’Ouvrages par Fibres Optiques (SOFO) interferometric sensors (dynamic and static systems) are long base sensors, measuring from 200 mm to 10 m. This system uses low-coherence interferometry to measure the lengths of a pair of single-mode fibers, a measurement fiber, and a reference fiber installed on the monitored structure. To capture the structure’s deformations, the measurement fiber is pre-tensioned and mechanically attached at two anchorage points, while the reference fiber is placed loosely in the same tube. Independent of the measurement base, the sensors have excellent long-term stability and precision of 2 μm. Since displacement information is encoded in the coherence of light rather than the intensity of the light, changes in the fiber transmission properties do not affect precision. Since the length difference measurements between the fibers are absolute, the reading unit and the sensors cannot be permanently connected.
- Fabry–Pérot interferometric sensors [354]: Fabry–Pérot sensors are point sensors that utilize optical cavities formed by two parallel reflecting surfaces to measure physical changes. Measurement is achieved by recording the Fabry–Pérot cavity’s length using white light interferometry. The sensors can be active or passive. Extrinsic Fabry–Pérot interferometers (EFPIs) are capillary glass tubes containing two partially mirrored optical fibers adjacent to each other with a remaining air cavity of a few micrometers between them. Two mirrors produce a back-reflected interference signal when light is launched into one of the fibers. Coherent or low-coherence techniques can demodulate this interference and reconstruct the changes in the fiber spacing. Capillary tubes are generally attached at their extremities with a space of 10 mm between them. Changes in the cavity can be correlated with strain variations between the two attachment points. Fabry–Pérot sensors are accurate, simple to use, versatile, and immune to environmental noise. They are typically used to measure strain, temperature, pressure, and displacements.
- Fiber Bragg grating (FBG) sensors [355]: FBG sensors are quasi-distributed (multiplexed) sensors that work on the principle of Fresnel reflection, where light traveling between media of different refractive indices can reflect and refract at the interface. A Bragg grating is a periodic alteration in the refractive index of the fiber core caused by the exposure of the fiber to intense ultraviolet light of 244 to 248 nanometers. A typical grating length is about ten millimeters. A grating can reflect light at wavelengths related to the grating period, while other wavelengths will be unaffected. Temperature and strain affect the grating period (length), so the spectrum of reflected light can be used to measure both parameters. For SHM of civil infrastructure, FBG sensors have been used to monitor tall structures, tunnel construction monitoring, and water pipe integrity monitoring. Figure 13 shows an overview of an FBG sensor. An application of the Bragg equation to light traveling along a Bragg grating core is given by:
  (1) $$\lambda_{B}=2n_{eff}P$$
  where $\lambda_{B}$: Bragg’s wavelength; $n_{eff}$: effective refraction index of the fiber core; *P*: Period of index modulation.
  Since the forward and backward propagating modes are coupled, the grating reflects a portion of the illuminated light and transmits the rest. Temperature and strain affect both $n_{e}ff$ and *P*, which is why the Bragg wavelength is sensitive to both factors. One of the most significant features of an FBG sensor is its capability to self-reference. Since the measurement values are encoded into wavelengths, which are absolute parameters, no calibration or reinitialization is required. FBG sensors have advantages over conventional sensors because they have gratings that can be multiplexed and positioned at different locations in the fiber, reflecting different wavelengths. Accuracy in the order of 1με and 0.1 °C can be achievable using the best demodulators.
- Distributed Brillouin and Raman scattering sensors [356]: In optical fibers, Brillouin and Raman’s scatterings result from the interaction of photons with localized material characteristics such as strain, temperature, and density. Due to different dynamic inhomogeneities in the silica fibers, Brillouin and Raman scattering effects have different spectral characteristics. Brillouin scattering operates backward, whereas Raman scattering operates forwards and backward. Emitting intense light at a known wavelength into a fiber causes minimal light to scatter from every location along the fiber. In addition to the original wavelength (i.e., the Rayleigh component), scattered light components are present with wavelengths higher and lower than the original signal (i.e., Raman and Brillouin components). These shifted wavelength components provide information on the local properties of the fiber, such as its strain and temperature. With a single unit, it is possible to measure thousands of points along the length of these fiber optic sensors. Raman scattering systems are typically accurate to ±0.1 °C and possess a spatial resolution of 1 m for measurement ranges up to 8 km. The best Brillouin scattering systems have a strain accuracy of ±20 με, a temperature accuracy of ±0.1 °C, and a measurement range of 30 km at a spatial resolution of 1 m.
- Multicore fiber sensors [357]: Multicore optical fibers are waveguides containing several cores. The cores are embedded in a common cladding and are sufficiently spaced to avoid the overlap of modes propagating. Multiple sensors can be accommodated in the same fiber cross-section owing to the independently propagating modes. Fan-outs or graded index lenses are typically used for coupling the different cores. Strain, temperature, vibration, and acoustic waves can be measured with these sensors. By interfering with different propagating lightwaves or wiring FBGs into the different cores, multicore FBG sensors can also be used to measure the curvature of different axes in the fiber cross-section. The cores can be accurately measured since they are located at various locations relative to the neutral bending axes. Inherent axial strain and temperature compensation also allow the accurate measurement of the local curvature.
- Microstructured optical fiber sensors [358]: Optical fiber communication systems and sensors have tremendous potential due to the recent invention of microstructured optical fibers. The cross-sectional geometry of these fibers is complex, including holes for both air and silica. The propagation lightwaves are confined either through a photonic bandgap effect (photonic crystal fibers) or an effective index contrast as in conventional optical fibers with air holes reducing the cladding index of refraction (holey fibers). Sensors applied to these fibers benefit from their complex geometry, providing new sensing opportunities and enhanced response characteristics. This fiber offers the following main advantages for sensing applications: (I) Since the fundamental mode propagates through less silica, the sensors have a near-zero temperature sensitivity; (II) single-mode fibers can be used across a wide wavelength range, allowing multiplexing of sensors with significantly different wavelengths; (IV) chemical sensing applications can be achieved by inserting gas into the air holes, allowing the propagating mode to interact with the gas over an extended surface area; and (V) by arranging the air holes, polarization maintaining (PM) fibers can be fabricated without inducing residual thermal stresses, which improves the stability of the birefringence when the temperature changes.
- Polymer optical fiber sensors [359]: An optical fiber made of polymer is known as a polymer optical fiber (POF). In the design of POFs, various optical polymers are used, including polymethylmethacrylate (PMMA), amorphous fluorinated polymer (CYTOP), polystyrene (PS), and polycarbonate (PC). POFs have a high elastic strain limit, a high fracture toughness, a high degree of flexibility in bending, a higher degree of sensitivity to strain than silica, and a negative thermooptic coefficient. Biocompatibility is another advantage of polymeric materials. A significant obstacle to using POFs as sensors is fabricating them. Generally, POF sensors are based on multimode POFs because of fabrication difficulties. Since multimode POFs are less expensive, they are also easier to connect, but their diameter is greater than that of single-mode POFs. Viscoelastic properties and extreme humidity sensitivity are also characteristics of POFs. A POF sensor may operate at a lower wavelength than an equivalent silica fiber sensor because polymers are highly attenuating in the near-infrared region. Some of the same measurement principles as silica optical fiber sensors are used to demonstrate multimode POF sensors, including intensity losses, backscattering, and time-of-flight measurements. Recently, new capabilities for POF sensors have been developed, including single-mode solid POFs and single-mode microstructured POFs. As a result of the emergence of these optical fibers, high-precision, large-deformation optical fiber sensing has become possible.
- Rayleigh scattering distributed sensors [352]: Distributed optical fibers based on Rayleigh scatter use backscattered light signals from naturally occurring impurities (scatters) in standard optical fibers to achieve sensing without introducing additional markers. This allows for sensors previously distributed over long distances to be replaced with a standard optical fiber, providing distributed measurements over large distances without needing expensive individual sensors. Rayleigh backscattering can be used to measure strain and temperature along the length of the optical fiber. The advantages of using Rayleigh scattering are high measurement rates, high spatial resolution, long-range, and higher efficiency, leading to higher signal-to-noise ratios.

Recent research in FOSs includes the following. Pant et al. [360] evaluated the use of distributed fiber optics to monitor a composite skin-stiffener joint of a helicopter tail boom. In addition to distributed FOSs, resistance wire strain gauges and flash thermography were used to assess the joint during quasi-static loading conditions. In [361], Li et al. examined different parameters, such as coating type and twist prestress, to improve the measurement sensitivity of distributed FOS in Rayleigh-Backscattering sensing (ODiSI-B by Luna Innovations) under aircraft operational and environmental conditions. Recently, an optical fiber grating temperature sensor inserted inside a lithium-ion Hardcase battery was proposed by Wu et al. [362] for long-term in situ temperature measurements. By combining laser-ultrasonic visualization with remote FBG-based sensing, Yu et al. [363] designed a high-temperature in situ damage diagnostic system. Because FOSs are made from silica glass, they have excellent heat resistance over 1000 °C, making them a potential high-temperature sensing technology. In AE detection, susceptible FBG sensors are widely accepted as fiber optic sensors. Since FBG diffraction gratings disappear at temperatures over 600 °C, Li et al. [364] proposed an FBG-based AE sensing system that uses a regenerated fiber Bragg grating (RFBG).

#### 4.4.3. Accelerometers

**Figure 14:**
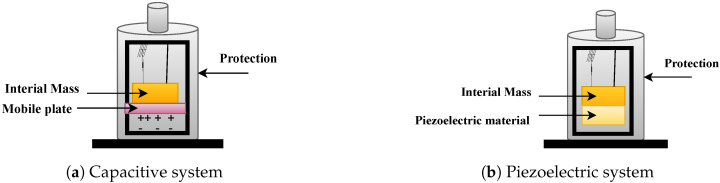
Accelerometer types.

Accelerometers are electronic sensors that measure acceleration forces acting on objects in order to determine their position and track their movement. In engineering, acceleration is the rate of change of an object’s velocity (velocity being its displacement divided by time). Acceleration forces come in two forms: static forces and dynamic forces, while a static force is constantly applied to an object (such as gravity or friction), dynamic forces are “moving” forces applied at different rates. In civil and aerospace structures, acceleration measurements are among the most common. Due to their well-understood nature and ability to provide information about a system’s local and global characteristics, they are a popular measurement quantity. In Figure 14, two types of accelerometer sensors, i.e., capacitive and piezoelectric, are shown.

The following section describes five accelerometer types widely used in health monitoring applications, i.e., piezoelectric, force-balance, capacitive, MEMS, and piezoresistance accelerometers:

- Piezoelectric: Piezoelectric accelerometers are the most widely used accelerometers in civil and aerospace applications. They typically measure acceleration in one of three modes: shear, flexural, or compression. Piezoelectric materials generate electrical charges in response to net forces acting on them. These materials can be classified as ceramic, polymer, and composite. Due to their electrical-mechanical transformation, piezoelectric materials act as both actuators and sensors at the same time. Since they contain no moving parts, they are long-lasting, can be used across a wide frequency range, exhibit good linearity across a wide dynamic range, and are robust and adaptable to various environments. Due to their minimum frequency requirements, these sensors are not capable of measuring static accelerations, unlike capacitive or piezoresistive accelerometers. In recent years, piezoelectric sensors have been incorporated into several SHM systems of civil engineering structures to measure acceleration, electrical impedance, elastic waves, and acoustic emission. The following lists some examples of SHM-related research and applications:
  – A piezoelectric oscillator sensor was developed in [365];
  – Impedance measurements were used for damage detection in [366];
  – Piezoelectric sensors were used for reference-free crack detection in [367];
  – Impedance-based self-diagnosis was investigated for piezoelectric sensors in [368];
  – A smart dual PZT transducer for damage detection was developed in [369];
  – A wireless sensor network SHM system based on piezoelectric sensors was investigated in [370];
  – A low-cost multifunctional wireless sensor node was developed using piezoelectric sensors in [371].
  The development of nanoscaled piezoelectric transducers has attracted increased interest due to their ultra-compactness and precision control. Future research on these sensor systems is proposed to focus on long-term ruggedness, miniaturization for increased flexibility, high-temperature applications, and high-strain and high-radiation conditions for permanently installed and embedded sensors.
- Force balance: These accelerometers employ active feedback control systems to control the position of a proof mass. Feedback is used to calculate the system’s acceleration keeping the mass stationary. During acceleration of the sensor housing, the proof mass stays stationary relative to the inertial frame of reference. Consequently, the proof mass moves away from its nominal position inside the sensor housing. An error signal is produced in the control system when a displacement sensor (often capacitive) detects this relative motion. Current flows through the force-generating element, balancing the force caused by acceleration. Based on the known proof of mass and system properties, acceleration can be calculated based on the current and force applied. Force balance accelerometers are often used for civil structure monitoring due to their excellent resolution at low frequencies, thermal insensitivity, and relatively nonlinear nature. There are, however, disadvantages of these types of sensors, including an expensive control mechanism and a limited bandwidth compared to other accelerometers. In [372], a force-balance-based accelerometer system was developed for measuring aerodynamic forces at impulse facilities using typical flight configurations. As part of the Indian Institute of Science’s hypersonic shock tunnel (HST2), Saravanan et al. [373] developed and tested a new three-component accelerometer force balance.
- Capacitive: A capacitive accelerometer determines acceleration by measuring the displacement of a proof mass relative to the sensor’s housing. Because the motion of the proof mass is relatively small in a capacitive accelerometer (less than 20 μm), it is typically suspended between two plates. Two capacitors measure small drifts and differentials between the mass and the top and bottom plates. Compared to piezoresistive accelerometers, capacitive accelerometers offer superior stability, sensitivity, and resolution, which makes them ideal for monitoring large structures. In contrast to piezo-electric accelerometers, they are somewhat sensitive to temperature and humidity fluctuations. Büsching et al. [374] provide a comprehensive review for the interested reader.
- MEMS: The technology of microscopic devices, particularly those with moving parts, is known as microelectromechanical systems (MEMS), also called microelectromechanical systems (or microelectronics and microelectromechanical systems). Micromechanics and microsystems are related technologies. MEMS devices generally range in size from 20 μm to 1 mm (i.e., 0.02 to 1.0 mm), and components range between 1 and 100 μm (i.e., 0.001 to 0.1 mm). Arrays of components (such as digital micromirror devices) can reach 1000 mm^2^. MEMS measure the linear acceleration of objects to which they are attached. The size and affordability of these devices allow them to be embedded in a wide range of hand-held electronic devices (such as smartphones, tablets, and video game controllers). Capacitive and ohmic switches are the two basic types of MEMS switches. MEMS switches with capacitive characteristics have moving plates or sensing elements that change the capacitance. Electrostatically controlled cantilevers control ohmic switches. As the cantilevers deform over time, ohmic MEMS switches can fail from metal fatigue of the actuator (cantilever) and contact wear. Whenever linear motion is required without a fixed reference, such as movement, shock, or vibration, MEMS accelerometers can be used. Lynch et al. [375] presented a detailed discussion of a high-performance, piezoresistive MEMS accelerometer with a planar structure. The performance of representative MEMS devices in SHM applications was presented by Parisi et al. [376], providing insight into the opportunities and capabilities of these devices.
- Piezoresistance: Piezoresistive accelerometers similarly measure stress to strain gauges. A force is applied to a piezoresistive material, and the change in resistance is measured after it is deformed. When pressure is applied to a piezoresistance accelerometer, its resistance increases. As a result of their high bandwidth, piezoresistive accelerometers are ideal for measuring high frequencies in a short period. Their low sensitivity makes them less useful for vibration testing. Interested readers can find a comprehensive review in [377].

#### 4.4.4. Electrochemical Sensors

An electrochemical sensor uses an electrode as a transducer element, and the tested sample acts as the analyte. A chemically selective layer (recognition element) is connected to the transducer providing real-time information about the system composition. Electrochemical sensors are classified as potentiometric, amperometric, or conductometric. By measuring the potential difference between two electrodes, potentiometric sensors can determine a sample’s composition. One of the most common potentiometric devices is the pH electrode. Detecting current flow with an amperometric sensor involves measuring the oxidation or reduction of an electroactive species between a reference electrode and a working electrode. An amperometric sensor consists of three electrodes: an auxiliary electrode made of conductive material (usually platinum), a counter electrode, and a reference electrode (usually platinum). The current is supplied to the working electrode by the counter electrode, which is larger than the working electrode. Conductometric sensors measure the conductivity of bulk materials or films at different frequencies. Humidity sensors often use mixed oxide conductivity sensors. Electrochemical sensors are highly dependent on the stability of their reference electrodes for quality measurements. Ciui et al. [378] presented a new electrochemical sensor capable of remaining electrochemically stable under high mechanical stress. Reinforced concrete structures are commonly monitored for corrosion using electrochemical sensors. Other applications of these sensors for concrete structures are assessing surface potential, noise analysis, polarization resistance, concrete resistivity, and galvanic current. Electrochemical sensors are further used in predictive maintenance, as discussed in [379]. Nowadays, these types of sensors are used in many aspects of modern life to detect physical, chemical, or biological parameters. Some applications include automobiles, planes, smartphones, and other technology media, as well as environmental and human health monitoring.

#### 4.4.5. Magnetostrictive Sensors

**Figure 15:**
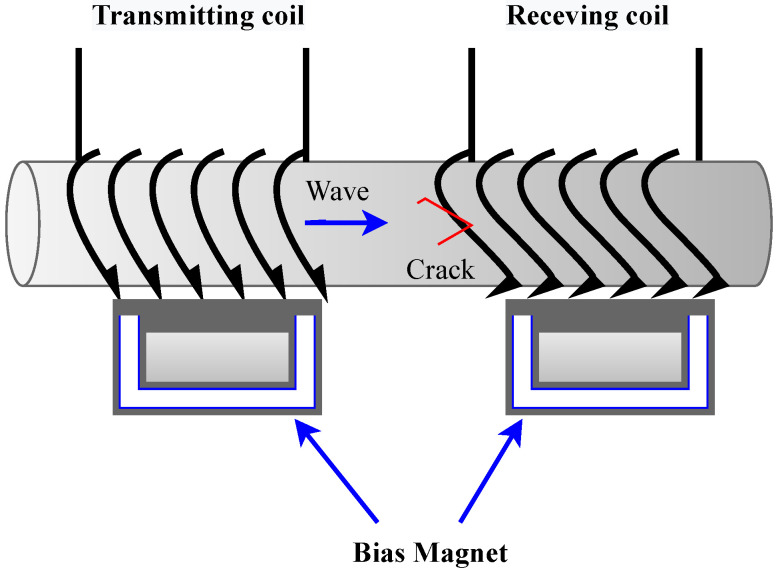
Principle of the magnetostrictive sensor.

Magnetostrictive sensors operate based on Joule, Villari, Wiedemann, or Matteucci effects; and are composed of five main components: waveguides, position magnets, electronics, strain pulse detection systems, and damping modules. Waveguide wires are typically enclosed in protective covers and attached to the measuring device. To generate a magnetic field, a current pulse is applied to the waveguide producing a sonic wave, which travels along the waveguide to a magnetostrictive material passing through the coil. Introducing stress into the magnetostrictive material alters the sonic wave’s permeability, changing the coil’s magnetic flux and voltage output. Voltage pulses are subsequently detected by electronic circuitry. Magnetostrictive sensors can be active, passive, or hybrid, depending on the usage of an excitation source. For passive sensors, changes in the magnetostrictive material properties are relied upon passively. In active sensors, the magnetostrictive element is internally excited to facilitate the measurement process. Hybrid sensors operate with an active magnetostrictive element that stimulates or changes another magnetostrictive element. A generic magnetostrictive system comprises three main components: the transmitting coil, the receiving coil, and the bias magnet. Figure 15 illustrates the working principle of a magnetostrictive sensor.

Current developments in magnetostrictive sensors use giant magnetostrictive materials (such as Terfenol-D), magnetostrictive amorphous wire, or thin films. Many applications use these sensor designs, including hearing aids, load cells, accelerometers, motion and proximity sensors, torque sensors, stress and force sensors, vibration sensors, magnetometers, flow meters, and more. Magnetostrictive sensors include magnetostrictive position sensors, magnetostrictive level transmitters, magnetostrictive force/stress sensors, magnetostrictive torque sensors, and magnetic field sensors. Non-contact magnetostrictive position sensors generate strain pulses along a waveguide using the momentary interaction between two magnetic fields. Unlike contact sensors, this type does not cause friction, is durable, vibration-resistive, and can operate for unlimited cycles. Its disadvantage is that the dead band cannot be reduced to zero on either side of the sensor. In applying magnetostrictive sensors to noisy environments, Zhang et al. [380] compared the white noise spectra produced by analog and digital circuits and found that the analog circuit produced a uniform and high spectrum of white noise. Thus, the researchers integrated the analog circuit into their magnetostrictive sensor design as the white noise source.

#### 4.4.6. Vibrating Wire Strain Gauge

A vibrating wire (VW) strain gauge works on the principle that a tensioned wire vibrates at a frequency that is proportional to the strain in that wire. Magnets and coils excite and sense the wire, which is tensioned between two end flanges. Different types of magnet/coil assemblies have been developed. In some models, the magnet/coil assembly is mounted outside a steel tube; while in others, it is integrated inside the tube. Most VW gauges are not affected by humidity; however, surface gauges should be protected from direct sun exposure. Every VW strain gauge has an inbuilt temperature sensor that enables temperature correction. No temperature correction is necessary in cases where a gauge is attached to a steel component since the gauge and the instrumented part have different thermal expansion coefficients.

VW strain gauges are typically large-size sensors (usually longer than 100 mm in length) and are commonly permanently attached or embedded in materials such as concrete. Surface strain gauges can be attached via welding, bolting, or bonding. Strain gauges that are embeddable can either be cast into concrete briquettes before placement or directly embedded in the concrete. To prevent stress discontinuity in the gauge area, large-diameter aggregates should not be placed near the gauge. The aggregate size should not exceed 1/5 of the gauge length within an envelope of 1.5 gauge lengths around the gauge. Neild et al. [381] presented a novel vibrating wire strain gauge capable of measuring strain in concrete elements. A comprehensive review of vibrating wire strain gauges is presented for the interested reader by Kuhinek and Zoric [382].

#### 4.4.7. Linear Variable Differential Transducers

Linear Variable Differential Transducers (LVDT) are used to measure displacement. These electromechanical devices convert the rectilinear motion of an object into electrical signals. The sensors are composed of a magnetically conductive shaft, known as the core, that is surrounded by a coil assembly. A liquid-level transducer contains a hollow metallic casing in which the shaft freely moves along the measurement axis. The coil assembly typically consists of a transformer with three windings—an upper and lower primary winding flank and a central secondary winding. Circuits are formed by connecting the secondary windings’ outputs. When an AC excitation is applied to the primary winding, the magnetically conductive core mediates the inductance current in the secondary windings. Secondary outputs are not affected by voltage when both secondary windings are equidistant from the core. With the movement of the core, a differential voltage induces the secondary output. As the core’s excursion from the center increases, the output voltage increases linearly. Joshi and Harle [383] provide details on various LVDTs and their application in civil engineering.

#### 4.4.8. Load Cell

Transducers that convert force into electrical output are referred to as load cells. These sensors measure mechanical forces, such as tension, compression, torque, or pressure, and translate them into digital recordings. Load cells can be classified into four types: piezoelectric, vibrating, pneumatic, and hydraulic load cells. The most common type of force sensor is a strain gauge load cell. Most modern industrial weighing systems use strain gauge load cells. An exception is specific laboratories that utilize precision mechanical balances. Pneumatic load cells are preferably used for safety and hygiene reasons, while hydraulic load cells are suitable for remote locations since they do not require power. For most industrial applications, strain gauge load cells offer accuracy within 0.03% to 0.25% full scale. Safizadeh and Latifi [384] developed a new method for diagnosing bearing faults by combing an accelerometer and a load cell.

#### 4.4.9. Foil Strain Gauge

Foil strain gauges detect length changes on the surface of a component as it experiences strain. They are typically fixed to structural components using adhesives or welding and are commonly used for experimental stress analysis. When higher accuracy is required, strain gauges that adhere to a surface should be selected; however, weldable strain gauges are preferred in difficult bonding conditions. Since bondable strain gauges require more surface preparation and adhesive curing time, their overall installation is more costly. Another type of foil strain gauge is a foil embedment strain gauge. This often type is used to measure structural strains.

Understanding the factors affecting the quality of the measurements is essential in ensuring the output reading represents the actual strain change in the material. Since foil strain gauges produce low-level voltage signals, they may be interfered with by electromagnetic or electrostatic fields, potentially leading to inaccurately interpreted signals. Hence, foil strain gauges are less attractive for bridge SHM where long distances need to be covered. Maintaining a stable reference for strain gauges (zero-stability) is vital for long-term measurements, especially in harsh environments, and additional gauge protection is often necessary. Dynamic measurements are problematic for foil strain gauges since noise filtering can alter the original signal. Tamura et al. [385] recently proposed to combine semiconductor strain gauges with metallic foil strain gauges to acquire higher dynamic range force measurements for robots.

#### 4.4.10. Tiltmeters

Tiltmeters are inclinometers that continuously measure very small changes in rotations, deflections, and deformations in structures such as walls, diaphragms, volcanoes, dams, or landslides. In addition to mechanical tiltmeters, electronic tiltmeters may incorporate vibrating wire or electrolytic sensors. With a highly sensitive instrument, it is possible to detect changes as small as one arc second. The different tiltmeter models include EL tiltmeters and EL beam sensors, tiltmeters with automatic data loggers, tiltmeters with SDI-12 interfaces, portable tiltmeters, uniaxial/biaxial tiltmeters, and wireless tiltmeters. Liu et al. presented a comprehensive review for the interested reader in [386]. There are many applications for tiltmeters and beam sensors, including:

- Monitoring of vertical rotation, deflection, and deformation in retaining walls.
- Monitoring differential settlements on railway lines.
- Assessing the stability of structures in landslide areas.
- Monitoring tunnel movement and convergence.
- Assessing the performance of bridges and struts.
- Inspecting critical structures and utilities affected by excavation/tunneling operations.
- Inspection of dams, piers, and piles for inclination and rotation.
- Monitoring volcanoes for structural changes or deformations.

#### 4.4.11. Laser Doppler Vibrometer

A Laser Doppler Vibrometer (LDV) combines a photodetector and a signal processor. LDVs work by reflecting a frequency-modulated laser beam onto an object, causing the reflected beam to shift in frequency (Doppler shift). A signal processor demodulates this shift and processes it to calculate the velocity of the object based on the difference in the reference frequency.

LDVs can be used for non-contact vibration measurements of a surface. By analyzing the Doppler shift of the reflected laser beam frequency due to the motion of the surface, the vibration amplitude and frequency are calculated. In general, an LDV produces a continuous analog voltage related directly to the target velocity component along the laser beam axis. Compared with similar devices such as accelerometers, LDVs have several advantages, including the ability to measure targets that may be difficult to reach or too hot for a physical transducer to be attached. LDVs measure vibrations without mass-loading targets, which is especially important for MEMS devices. Darwish et al. [387] used LVD and convolutional neural networks for non-contact vibroacoustic object recognition.

#### 4.4.12. Acoustic Emission Sensor

**Figure 16:**
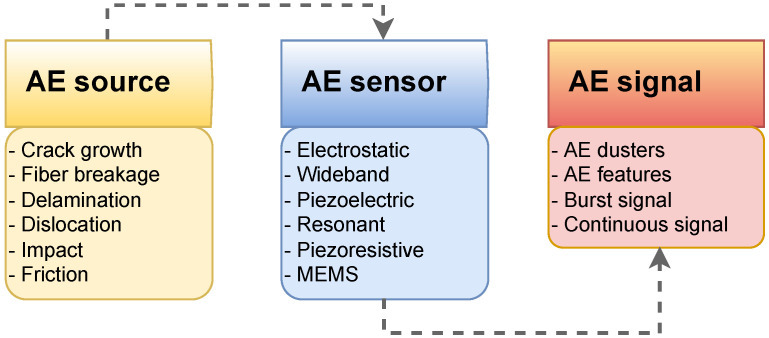
AE sensor bridging AE source and AE signal in a simple acoustic emission sensing chain.

An AE sensor measures high-frequency energy signals that occur when the internal structure of a material undergoes irreversible changes. Acoustic emissions are caused by the rapid release of localized stress energy, for example, by the formation of cracks, and consist of inaudible ultrasonic signals. Depending on the propagating material, this effect is also termed structural noise. Figure 16 presents different types of AE sources, sensors, and signals for damage detection. In an AE sensor, the mechanical signal generated by an AE source is converted into an electrical signal, the AE signal, by propagation through the material.

In AE applications, frequencies typically range from 20 kHz to 1 MHz. Depending on the sensor’s frequency response, there are two qualitative types: resonant and wideband. In the 20–500 kHz range, resonant AE sensors have a high quality (Q) factor (low damping), resulting in a narrow bandwidth and high sensitivity. A wideband AE sensor has a low Q factor, high damping, wide frequency range, and low sensitivity. AE sensors can be embedded in structures to identify and locate damage using modal analysis. In conventional AE sensors, bulky piezoelectric ceramics are used. A current trend in AE manufacturing is a move from manual towards automated mass manufacturing with increased reliability, lower costs and smaller sizes. Combining AE sensors with other sensor systems, such as MEMS and piezoelectric transducers, is an active field of research. A MEMS-AE sensor is typically designed as a resonator to amplify the signal-to-noise ratio resulting in a smaller size sensor than a conventional AE sensor. A piezoelectric MEMS-AE sensor consists of a resonating silicon microstructure and a thin piezoelectric layer mounted on ceramic. MEMS-AE devices are significantly smaller and lighter than conventional AE sensors. The design and manufacturing stage of an ultrasensitive AE sensor optimized to detect partial discharges in power transformers is described by Sikorski [388]. In [389], three types of AE sensors were used to detect defects in angular contact ball bearings. Caso et al. [390] used AE sensors to monitor shaft misalignment in low-speed gears.

#### 4.4.13. Temperature Sensors

Temperature changes can affect the physical properties of materials and consequently influence the characteristics of parameters used for SHM of civil structures. For example, an increase in temperature can cause an increase in structural strength, changing the modal parameters of a structure [391]. Analyzing the temperature can provide information on internal conditions, such as cracking, fatigue, and yielding. Further, since some sensors are sensitive to temperature, measuring the temperature and potentially applying temperature corrections is essential. Various sensors are used for this purpose, including thermocouples, resistance temperature detectors (RTD), thermography, biomaterial temperature sensors, fiber optic, pyroelectric thermometers, and electrical resistance thermometers.

Temperature variations in a measured signal can sometimes be mistaken as damage [392]. Several studies have been published on the effects of temperature variation on measurement signals from SHM systems [391]. Dhingra et al. [393] developed a Bragg grating-based sensor to monitor the health state of civil structures at different temperatures. According to simulation results, a linear shift in Bragg wavelength was observed when strain and temperature were independently increased. Furthermore, if temperature and strain were both increased simultaneously, a directly proportional relation was observed in Bragg wavelength. The study confirmed the enhanced performance of the developed sensor with potential applications in civil, military, and bio-medical fields.

An evaluation methodology for temperature compensation in monitoring systems was presented by Caspani et al. [394]. The focus was set on condition-state parameters characterizing the long-term response trend, such as creep and shrinkage effects and tension losses. The researchers derived an equation using temperature-compensated response measurements to estimate uncertainties in the long-term response. It was further shown that the condition-state uncertainty is affected by the measurements, model uncertainties, sampling frequency, start date, and period of the monitoring activity.

Ideally, sensors should operate independently of environmental and operational variations (EOVs). Several studies have been published researching the effects of temperature variation on sensor measurement signals [11]. In Table 25, we list different advanced sensors specifying their applications, year of development, and maximum operating temperature.

Depending on the application, various temperature sensors exist with different characteristics. There are two basic types of temperature sensors:

- Contact temperature sensors: For these sensors, the object being sensed must be in physical contact with the sensor, and temperature changes are monitored via conduction. They can be used on solids, liquids, and gases for various temperatures.
- Non-contact temperature sensors: Sensors of this type monitor temperature changes via convection and radiation. Radiation detectors can detect liquids and gases that emit radiant energy in convection currents or infrared radiation transmitted from objects (the sun).

Temperature sensors can further be subdivided into electro-mechanical, resistive, and electronic. In the following, two common temperature sensors used for SHM are discussed:

- Resistive temperature sensors: These types of sensors work on the principle that resistance changes with temperature. Most resistive temperature sensors are either metallic sensors or thermistors. Metallic sensors consist of platinum wires wrapped around a mandrel and are covered with protective coatings or enclosed in protective housings. Platinum resistance changes linearly with temperature. Hence, Wheatstone bridge circuits can be assessed by installing one of these sensors in one circuit arm. A highly nonlinear relationship exists between a thermistor and resistance temperature. To overcome this challenge, matching pairs of thermistors are used to offset their nonlinearities. In general, thermistors are more accurate than metallic temperature sensors, but their operation range is smaller. Ceramic semiconductors can also be used as thermometers due to their change in resistance. It is noted that there are fundamental limitations to all resistive sensors. Even though the current used to operate these sensors is very small, it creates heat, leading to inaccurate temperature readings.
- Vibrating wire temperature sensors: The operation of vibrating wire temperature sensors is similar to that of VW strain gauges. In A VW temperature sensor, changes in the temperature affect the frequency at which the wire vibrates or resonates. The sensor produces a voltage proportional to the temperature reading or displays the temperature in temperature units. Since the VW temperature sensor is enclosed in a cylinder, it cannot physically contact the testing material. Therefore, no special precautions are necessary for strain effects on sensor readings.

#### 4.4.14. Next-Generation Sensing Techniques

A new generation of intelligent non-contact monitoring systems for civil infrastructure arrived with the development of computationally efficient smartphones, drones, inexpensive high-resolution cameras, and robotic sensors. The growth of next-generation measurement technology for SHM is driven by the need to advance and develop alternative methods for efficient sensing systems. Recent advancements in sensing and robotic technology led to the development of next-generation sensing techniques that outperform traditional contact-based sensors, including wired and wireless sensors. Next-generation sensors are used to monitor various systems, including drones, robotic sensors, wireless sensors, cloud services, GPS, video cameras, machine vision, smartphones, and high-speed cameras. These sensors and applications are briefly described below.

- Unmanned Aerial Vehicles (UAVs): Inaccessible surfaces or the requirement to install a large number of sensors limit the affordability of traditional contact-based sensors. Recent advances in non-contact measurement technology were made possible with UAVs, namely, drones. UAVs are aircraft without pilots, crews, or passengers. Rapid developments in control theory, computing capabilities, robotics, communications, and automation technologies provide the platform for the wide variety of applications of UAV technology in SHM systems. UAVs are now equipped with lightweight cameras to take pictures and estimate the structure’s global and local health. Most UAVs are composed of a navigation system with visual servoing, a global positioning system (GPS), and a vision system typically consisting of an out-of-the-box camera (e.g., an infrared camera, optical sensor, or laser detection and ranging (LADAR)). A navigator on the ground can control the aircraft remotely by controlling its in-flight data acquisition and post-flight image processing. Recent UAV applications include traffic monitoring, construction inspections, surveying, and health monitoring of roads, bridges, pipelines, and buildings, particularly in the transportation sector. In addition to reducing workplace accidents, UAV sensors reduce logistics and working hours. Compared to satellite images, they provide excellent temporal and spatial resolution, making them suitable for monitoring inaccessible areas. UAV sensors can provide 3D information about structures which can be used for large-scale system monitoring and management. Ortiz et al. [409] studied the use of UAVs for heritage site surveillance. An algorithm called CornerHarris was applied by Cho et al. [410] to detect cracks using UAVs. The researchers used Haar-like features and converted color images to greyscale to identify the damage. UAV-based crack detection of a concrete bridge was investigated by Reagan et al. [411].
- Vision-based sensors: In vision sensors, cameras are typically used to capture images and determine various characteristics of an object, including its position, orientation, and surface composition. Vision-based sensors do not require physical contact with the object or long-wired transmission networks providing benefits in cost reductions, ease of use, a wide range of applications, and improved reliability. A critical difference between image inspection systems and these sensors is that the camera, light, and controller are integrated into one device, simplifying installation and operation. In the last few years, vision-based sensors have been studied for their use in system identification. Despite presenting a significant step forward in innovation, some challenges exist, providing an exciting opportunity for further research. Lighting in the workspace, for example, may limit the measurement accuracy with the tracked object needing repositioning. As a result, modal parameter estimation may be inaccurate due to the incorrect mapping of the reference system. Sony et al. [412] used vision-based sensors for crack detection in real-life networks applying algorithms that can isolate the tracking point regardless of the picture’s luminosity. Helfrick et al. [413] investigated using stereo cameras to detect damage caused by curvature changes in 3D digital image correlation (DIC). According to Huňady et al., [414], a Q450 Dantec dynamics camera was used to estimate the damping of steel plates at 1000 frames per second. Using video recordings, Yang and Yu [415] developed a vision-based method to monitor vibrations such as velocity and displacement.
- Smartphones: Analytical devices are becoming increasingly common in our daily lives for various purposes, including communication, personal care, food allergen detection, clinical analysis, and environmental monitoring. Smartphones, the most popular state-of-the-art mobile devices, are equipped with various sensing technologies such as GPS, accelerometers, and gyroscopes, which can be used to assess the condition of structures. Modern smartphones are embedded with a growing variety of physical-chemical sensors, including ambient light sensors, magnetometers, proximity sensors, accelerometers, microphones, gyroscopes, GPS, touchscreen sensors, fingerprint sensors, pedometers, barcodes/QR codes, barometers, heart rate monitors, thermometers, air humidity monitors, and Geiger counters. Rapid developments in integrated smartphone sensing technologies resulted in improved sensing capabilities, cost-effectiveness, flexibility, durability, smaller size, and reduced weight.
  Since 2010, smartphones have been increasingly used in SHM applications using their wide variety of sensing capabilities–all in one device–and benefiting from their increased storage capacities, computing power, and easily adaptable software. Dashti et al. [416] used accelerometers in four iPhone 3GS and three iPod touchpad devices to collect vibration measurements from a 3D shake table test to monitor earthquake-induced ground motion. Cimellaro et al. [417] developed a mobile application that can run on iOS, Android, and BlackBerry platforms to assess earthquake damage. Zhao et al. [418] presented the development of a cable force measurement software based on the iPhone (for iOS 7.0 or higher platforms) called Orion Cloud Cell, which provided benefits in ease of use, convenience, accessibility, and time efficiency.
- Wireless sensors: In wireless sensing, sensory information is transmitted using wireless transceivers, avoiding the need for cost- and labor-intensive cabling. Wireless sensors do not process data locally, and hence, they require very little power. Energy is either supplied from an external power source such as batteries, or the sensors are self-powered, drawing power from sources including vibrations (piezoelectric energy harvesting), temperature gradients (thermoelectric energy harvesting), or radio waves (RF energy harvesting). Wireless sensing technologies have been developed to monitor physical or chemical conditions such as vibration, sound, temperature, humidity, pressure, sound, or pollutants. Several wireless sensors can be grouped to form a wireless sensor network (WSN) of spatially dispersed sensors. In a typical network, each sensor shares data through nodes that consolidate information or through a gateway that serves as a local wireless access point and router. Wireless sensors transmit very light data loads, which can be supported on low-speed networks.
  There are two types of wireless sensors: passive sensors and active sensors. A passive sensor measures a physical or chemical quantity passively based on the system’s state and comprises three functional subsystems: a sensing interface, a computational core, and a wireless transceiver. In contrast, active sensors generate excitation signals and then sense the system’s response. An additional subsystem (the actuation interface) generates the signal in the active sensors. Sensing transducers can be connected to wireless sensors through an interface that converts the analog outputs of the sensor to digital representations. Following the collection of measurement data by the sensing interface, local data processing and computation are carried out by a computing core. Consequently, less computational load is placed on the central data processing system. This task is accomplished with the help of a microcontroller that allows measurement data to be stored in random access memory and data interrogation programs to be held in read-only memory. A wireless transceiver is required to transmit and receive data from other wireless sensors and for their transmission to remote data repositories. Lastly, an actuation interface allows wireless sensors to interact directly with physical systems. A digital-to-analog converter (DAC), which creates continuous analog voltage output from the microcontroller, is at the core of an actuation interface.
  Despite the many benefits of wireless sensing, there are significant challenges associated with battery-powered wireless sensors, including their energy consumption, hardware design, cost, size, communication range, and risk of data loss. Energy-harvesting sensors are being developed to address power consumption issues in wireless sensors and new sensing techniques, such as radio-frequency identification (RFID) sensors. In [183], continuous monitoring of material integrity was achieved through ultrasonic-based NDT combined with wireless sensors. A wide range of critical issues related to WSN application in SHM was discussed by Wang et al. [419], including sensor integration, sampling frequencies, transmission bandwidth, real-time capability, and frequency of wireless transmitters. A study conducted by Fu et al. [420] examined the impact of wireless communication technology known as GPRS (General Packet Radio Service) on hydraulic engineering NDT techniques. Zhang et al. [421] proposed a novel passive wireless sensor based on ring dielectric resonators for position-insensitive crack monitoring.

Table 26 summarizes our extensive review of papers from 2000 to the present related to the use of advanced sensors in SHM.

### 4.5. Data Analysis for SHM

In any SHM system, the analysis of measurement data is the second essential process for capturing structural characteristics using sensory systems. The recorded raw data typically undergoes a process of data acquisition, signal conditioning, data transfer, data storage, signal processing, and data interpretation for damage detection. Many data analysis approaches and algorithms have been developed and are constantly further advanced depending on the type of sensors and measured data. The fast-growing field of data science, with rapid advances and innovations in artificial intelligence (AI) and data mining, led to a transformation and renewal of data analysis methodologies for SHM, while data analysis techniques, such as traditional signal processing, are applied to datasets to execute and test models and hypotheses, regardless of the amount of data, AI methods, such as deep learning, are used to uncover hidden patterns in large volumes of data [460]. The following sections present an overview of recent developments in signal processing techniques and the application of deep learning, the most progressive AI technology, in SHM systems.

#### 4.5.1. Signal Processing Methods

Complex processes characterize the structural response to time-variant loading, and hence, one of the most challenging aspects of SHM is the extraction of damage features via signal processing [461]. A signal processing algorithm for SHM must be able to deal with noise and complexities embedded in the measurement signal while identifying the features of interest. The objective of signal processing is defined as [462]: the extraction of features from the recorded data for (1) identification of the status of the system (damaged or healthy), (2) localization of damage, (3) quantification of damage, and (4) identification of the damage type.

Several methods and mathematical models have been proposed to analyze signals from sensor systems associated with the time-frequency or time-frequency domain. Some examples of signal processing techniques include Kalman Filter (KF), statistical time series (STS) models, fast Fourier transform (FFT), wavelet transform (WT), short-time FFT (SFFT), S-transform (ST), Hilbert transform (HT), fast ST (FST), Hilbert-Huang transforms (HHT), blind source separation (BSS) and multiple signal classification (MUSIC). System characteristics can be determined by employing these techniques, and damage features derived [463]. In Table 27, we summarize the strengths and shortcomings of the methods mentioned above for convenient selection and utilization.

#### 4.5.2. Deep Leaning in SHM

**Figure 17:**
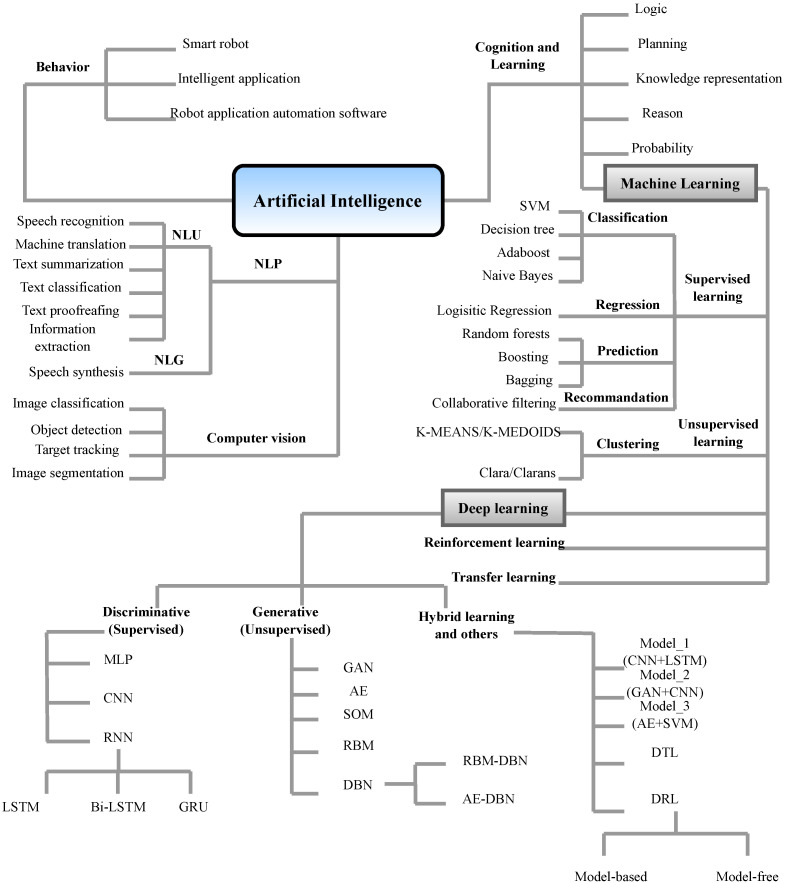
A taxonomy of AI, ML, DL.

Recent advancements in the data science fields of data mining, artificial intelligence (AI), and machine learning have opened new possibilities for developing next-generation SHM systems [12,464]. Artificial Intelligence (AI) systems such as Machine Learning (ML) [465,466,467] or Deep Learning (DL) [468,469] techniques have gained much traction, and the research community has paid considerable attention in recent years to investigating its integration in SHM systems. AI techniques, such as artificial neural networks (ANNs), simulate the behavior and intelligence of humans, while ML is a group of learning techniques that can automate analytical model building and incorporate information or experience into a machine or system [289]. DL architectures are based on ANN techniques and use multiple layers of neurons to extract higher-level features from large data sets progressively and can model complex non-linear relationships. The term “Deep” in deep learning is defined as the concept of multiple levels or steps through which the information is processed to form a data-driven model. DL is one of the most progressive and popular technologies of AI systems incorporating feature extraction and classification and can be applied to build intelligent systems and automation models. Figure 17 shows the position of DL as a branch of ML and AI.

Below, the primary reasons for the rapid advances and profound interest in DL-based SHM are outlined:

- Advances in Data Science (DS): A few decades ago, the terms “Data Science” and “Data Engineering”, the core of data-driven applications, were not yet defined. Recent advances in AI led to DL algorithms with highly advanced capabilities in data engineering tasks such as outlier detection, feature extraction, and data recovery. These capabilities are essential for any SHM system and provide the complementary base of DL and SHM.
- Advances in computer hardware and software (CHS): Recent improvements in multi-core processors led to sophisticated graphics processor units (GPU) expediting the processing of deep neural networks training in cloud-based platforms online.
- Advances in Transfer Learning (TL): Latest innovations in transfer learning attracted extensive attention toward DL-based SHM due to their improved generalization and time-saving capabilities. Pre-trained networks, like VGG, ResNet, and AlexNet, opened new research fields and increased DL-based applications in SHM.
- Advances in cloud-based computation and big data (CC—BD): Rapid advances in cloud-based computing and wireless technologies, combined with a trend of decreasing costs for sensors, portable devices, and cameras, facilitated the sensor deployment and facilitated wireless data transfer into cloud-based computing systems, making autonomous monitoring regimes on complex infrastructures feasible.

DL models can be categorized into four basic groups:

- Supervised learning: Learning from labeled training information to predict results for unseen data. Classification and regression are the most popular applications of supervised learning.
- Unsupervised learning: Labelled data are not required. Data are clustered by finding hidden patterns and relationships among data points.
- Semi-supervised learning: Refers to a type of training problem where the data set is composed of a small volume of labeled data and a large volume of unlabeled data.
- Reinforcement learning: This scheme, which is situated between supervised and unsupervised learning, provides an “agent” that has the capability to learn from its surrounding environment.

As stated above, there is a noticeable increase in research incorporating DL in SHM algorithms. Convolutional Neural Networks (CNNs) [470] and Recurrent Neural Networks (RNNs) [471] are among the most commonly used algorithms in DL-based SHM models. The most advanced DL strategies that are used in SHM systems include Generative Adversarial Network (GAN), Adversarial Autoencoder Network (AE), Variational Auto-Encoder Network (VAE), VAE-GAN Network, Adversarial Variational Bayes (AVB) Network. Adversarially learned inference (ALI) and Bi-directional GAN (BiGAN) Network. Table 28 reviews recent papers using DL-based models for SHM.

## 5. Future Directions

Based on the extensive research undertaken for this review article, we summarize the most important future research directions as follows:

- The newest generation of sensing systems incorporates recent innovations in sensor technologies such as intelligent materials, active sensing, wireless data transfer, and deep learning, while some of these novel techniques have been applied to real structures, many have only been studied under research conditions, and they must be further explored in real-life environments for their benefits and challenges to be fully assessed and understood.
- NDT and SHM of components exposed to high temperatures (>650 °C) is a field of increasing importance. Their implementation, however, poses significant challenges due to the harsh high-temperature environments. The development of advanced sensors suitable to these environments is, therefore, an essential field of future research.
- Despite significant progress in sensing developments, many challenges remain demanding further research efforts. The next generation of smart structures is aimed to incorporate smart materials with embedded sensing power that consume only little energy or are self-powered, resist noise and environmental variations, and are cost-effective and eco-friendly.
- Novel vision-based sensors need to be insensitive to light conditions, demanding the development of improved algorithms for image processing.
- Self-sensing materials are an exciting field of research investigating strategies such as embedding fiber-optic and piezoceramic sensors in a structure’s critical components. Future research directions are aimed at developing self-sensing materials that are able to instantly identify any material changes induced by damage in the nano- or microstructure.
- Further research is needed in the field of deep learning algorithms as potential replacements or additions to traditional data analysis approaches for intelligent pattern recognition for damage identification and classification.
- Big data-driven product design is a hot topic with many ongoing developments and applications related to the Internet of things (IoT).
- Digital twin technology, which analyzes sensor data using AI algorithms, is a cutting-edge technology linking the physical and virtual worlds. More research is needed to exploit the capabilities of digital twin technology fully.
- Existing sensor technologies, such as cameras, microphones, inertial measurement units, etc., are widely used for various applications, but their high-power consumption and battery replacement remain a concern. Further developments are needed on self-powered sensors, such as using triboelectric nanogenerators (TENGs) that provide a feasible platform to realize self-sustainable and low-power systems.

## 6. Conclusions

Our aging infrastructure is considerably burdened by current economic growth and population expansion. To assess the structural integrity of these critical structures, various types of Non-Destructive Testing (NDT) and Structural Health Monitoring (SHM) methods have been developed to assist engineers and managers in identifying, evaluating, and prioritizing structural rehabilitation and thereby prevent catastrophic failures resulting in economic loss and potential human death. The rapid evolution of sensor technologies suitable for NDT and SHM applications resulted in the availability of vastly complex sensing systems presenting challenges for engineers in selecting the most appropriate sensing technique for individual applications. This paper aims to provide a state-of-the-art review of articles focusing on technological advancements in sensing systems used for NDT and SHM in civil engineering applications. According to the authors’ knowledge, this is the first article presenting a systematic and comprehensive overview of the latest developments in advanced sensor technologies. Working principles, technical specifications, and applications of various methods are presented, and their advantages and disadvantages are discussed. Conventional sensor techniques are also included as they are well understood and provide established technologies with known and reliable benefits and limitations.

## Figures and Tables

**Table 1:** Examples of recent research on advanced sensor technologies for NDT and SHM applications (between 2000 and 2023).

Sensor Type	NDT/SHM Technique	Application	Damage Type	Year	Ref.
Fiber optics	Acoustic emission	Carbon-fiber reinforced laminate composite	Matrix cracking/fiber fractures	2000	[20]
Micro-architectured synthetic nervous system	Acoustic emission	Composite and heterogeneous material systems	Cracks	2001	[21]
Fiber optics	Real-time monitoring (RTM) flow monitoring system	Composite aerospace structure	Cracks	2002	[22]
Ultrasonics	Lamb wave method	Carbon fiber-reinforced polymers (CFRP) cross-ply laminates	Transverse cracking and delamination	2003	[23]
Laser vibrometry	Acousto-ultrasonic waves	Aluminum plates	Fatigue cracks	2004	[24]
Magnetic field	Eddy current probe	Austenitic stainless steel	Fatigue damage	2005	[25]
Fiber Bragg gratings (FBGs)	Ultrasonic waves	Cross-ply CFRP	Cracks	2006	[26]
Pulsed laser	Ultrasonic visualization	Polymer-matrix composites (PMCs)	Delamination	2007	[27]
Radar	Far-field airborne radar (FAR)	Glass fiber reinforced polymer (GFRP)	Delamination	2008	[28]
Piezoelectric	Ultrasonics	Ceramic composite plate	Cracks	2009	[29]
Optical fiber	Optical backscatter reflectometer (OBR) technique	Bolted joints	Cracks	2010	[30]
Acoustic emission	AE technique	Concrete materials	Cracks	2011	[31]
Ultrasonics	Shearography	Composite laminates	Delamination	2012	[32]
Acoustic	Digital image correlation	PMCs	Cracks	2013	[33]
Ultrasonics	Ultrasonics	GFRP	Cracks	2014	[34]
Acoustic	Acoustic emission	Railway	Cracks	2015	[35]
Piezoelectric transducer (PZT) sensors	Bayesian method	Plate	Cracks	2016	[36]
Piezoelectric actuators	Imaging technique	Aluminum plate	Cracks	2017	[37]
Actuator-sensor	Ultrasonics	Wind turbine blade	Cracks	2018	[38]
Ultrasonics	Ultrasonic pulse velocity (UPV) technique	Reinforced concrete	Debonding	2019	[39]
Ultrasonics	Mobile ultrasonics	Fiber-reinforced plastics (FRP)	Cracks	2020	[40]
Piezoelectric	Electromagnetic wave (EMW) technique	CFRP composites	Fiber breakage	2021	[41]
Ultrasonics	Ultrasonics	Reinforced concrete structure	Cracks	2022	[42]
Ultrasonics	Ultrasonics	Concrete surfaces	Initial freeze-thaw damage	2023	[43]

**Table 2:** Civil structure assessment using NDT and SHM techniques for various aims.

Structure	Monitoring Aims
Dams	- Footing settlement. - Stress and vibration monitoring. - Temperature monitoring during curing.
Bridges	- Stress monitoring of long spans with hundreds of sensors. - Tracking behavior in high-stress conditions. - Embedded sensors in concrete beams and pilings. - Long-term stress and vibration monitoring. - Surface-mounted sensors on steel components in expansion joints. - Embedded or surface sensors on cables for suspension structures. - Strain sensing on steel girders.
Tunnels	- Monitoring of stresses and strains. - Settlements. - Crack detection and monitoring.
Hillsides	- Detection and prediction of possible landslides. - Monitoring of gradual shifts in rock and soil.
Reservoirs	- Monitoring of river levels and flows. - Improvement of flood control. - Accurate monitoring of reservoir water levels to control the dam’s flow.

**Table 3:** Exclusion and inclusion criteria for selecting the reviewed articles.

Inclusion Criteria	Exclusion Criteria
Title, abstract, or keywords include the following search keywords: “Structural Health Monitoring” “Non-Destructive Testing” “Damage Identification Methods” “Advanced Sensor Technologies”	Papers published before 2000. Duplicated papers (only one paper included). Articles unrelated to SHM or NDT content. Non-English papers. Not peer-reviewed papers. Poor quality papers.

**Table 4:** Applications fields of various NDT methods.

NDT Method	Application Fields	Refs
Ultrasonic testing (UT)	Aerospace industries; Material research; Quality assurance; Bridges; Gas trailer tubes; Weld inspection; SHM	[62,63]
Neutron imagine (NI)	Civil Engineering; SHM; Aerospace industries	[64]
Acoustic emission (AE)	Aerospace industries; Machining; Civil Engineering; Automobile industries; SHM	[65]
Nonlinear acoustics (NLA)	Medicine; Machining; Automobile industries; Civil Engineering; Aerospace industries; SHM	[66]
Digital image correlation (DIC)	Aerospace industries; Automobile industries; Medicine; Civil Engineering; Machining; SHM	[67]
X-ray radiography and X-ray tomography (XRI)	Civil Engineering; SHM	[68]
Terahertz (THz)	Aerospace industries; Civil Engineering; SHM	[69]
Resistivity	Civil Engineering; SHM	[70]
Infrared thermography (IRT)	Medicine; Civil Engineering; Aerospace industries; Optimizing processes; Surveillance; SHM	[71]
Shearography (ST)	Machining; Aerospace industries; Civil engineering; SHM	[72]
Neutron imagine (NI)	Civil Engineering; Aerospace industries; Automobile industries; SHM	[73]
Eddy current testing (ET)	Aerospace industries; Civil Engineering; SHM	[74]

**Table 5:** Recent NDT studies and their applications.

Application	NDT(s)	Description	Refs
Component manufacturing	Ultrasonics with deep learning	The application of deep learning techniques is studied on ultrasonic NDT for porosity evaluation of additively manufactured components.	[75]
Oil and natural gas pipelines	Eddy current array	Laboratory study on steel pipeline specimen using Eddy Current Array (ECA), a recent advancement of the conventional Eddy Current (EC) method.	[76]
Ship and marine structures	Ultrasonics	Time-domain waveforms are analyzed to compute the time-of-flight data to determine the thickness of coating materials.	[77]
Fiber composites	Acoustic emission with micro-computed tomography	Acoustic emission technology are evaluated for monitoring bending damage of 3D printed continuous fiber composites in real-time.	[78]
Fire-damaged reinforced concrete buildings	Ultrasonics and mechanical impact	Gene algorithm techniques are combined with ultrasonic pulse velocity and rebound hammer to model the temperature in fire-damaged reinforced concrete buildings.	[79]
Prestressed reinforced concrete	Various NDTs	Potential applications and advancements of NDT technologies for infrastructure construction.	[80]
Various	Various NDTs	This special issue presents novel approaches for non-destructive testing and evaluation.	[81]
Laminate composites	Various NDTs	Several NDT methods are compared. Particular focus is placed on identifying zones with bonding defects and identifying the most appropriate techniques.	[82]
Various	Ultrasonics	Correction method based on ultrasound propagation and acoustoelastic theory is presented. Further, the quantitative impacts of the stress field are studied.	[83]
Timber	Various NDT	The influence of the number and locations of measuring points on predicting the flexural modulus of aged wood is studied for various NDTs.	[84]

**Table 6:** Common NDT technologies, inspection types, and probe styles used for steel.

Inspection Type	NDT Technology	Common Probe Style(s)	Refs
- Flaw detection - Crack detection - Void detection - Inclusion detection - Weld Inspection	- Eddy Current - Ultrasonics	- Angled/curved tip - Surface probe - Tubing probe - Bolt hole probe - Inner diameter probe - Angle beam - Immersible, waterproof	[89,139,140,141,142,143,144]
- Material thickness - Section thickness - Wall thickness	- Ultrasonics - Eddy Current	- Dual - Wheel probe - Immersible - Bolt hole probe - Inner diameter probe - Immersible, waterproof	[1,115,145,146]
- Corrosion detection - Erosion detection - Pitting detection - Other irregularity detection	- Eddy Current - Remote Field Testing	- Pencil probe	[147,148]
- Coating thickness - Conductivity	- Inductive - Eddy Current - Magnetic/Hall Effect	- Angled/curved tip - Pencil probe	[149,150,151,152]

**Table 7:** Common NDT technologies, applications, and benefits used for concrete, wood, composites, and masonry.

Material	NDT Technology	Application(s)	Benefit(s)	Refs
Concrete	Windsor probe	- Determination of concrete compressive strength	- Accurate, efficient, and enhanced - Quick, convenient, and economical procedure - No accidental discharge or recoil	[153] [154]
Concrete	Concrete test hammer	- Assessment of in-place uniformity of concrete - Detection of deteriorated concrete and regions of poor quality	- Affordable equipment - Straightforward procedure	[155] [156]
Wood	Drill resistance	- Detection of internal voids and advanced decay	- Affordable and portable equipment	[157] [158]
Wood	X-ray technique	- Identification of defects using density measurements	- High-reliability - Ease of interpreting results	[159] [160]
Composites	Electromagnetic testing	- Fault detection - Fracture evaluation	- Detection of both external and internal defects - Suitable for inaccessible zones	[161] [162]
Composites	Shearography testing	- Detection of epoxy matrix deficiency or excess - Detection of disbonds, un-bonds, and delamination	- Less susceptible to noise than many other types of NDTs - Fast, accurate, real-time information about internal material	[163] [164]
Masonry	Flat jack testing	- Estimation of mechanical properties - Determination of in situ stress for structural evaluation	- Adequate accuracy - Versatile practicality	[165] [166]
Masonry	Impact Echo testing	Detection of stress wave propagation and flaw	Accurate	[167]

**Table 8:** Advanced ultrasonic sensors.

Type	Details
Self-contained sensors	Devices with an ultrasonic sensor and controller integrated into one unit. The range covers 2 inches to 40 feet.
High-accuracy sensors	The range of precision distance measurements are from 3 inches to 16 inches.
Close-range sensors	For close measurements ranging from 0.5 inches to 30 inches.
Intrinsically safe sensors with accessories	Ultrasonic sensors for use in hazardous environments. Ranges are from 4 inches to 18 feet.
Remote sensing heads	The sensor is a remote unit connected to the controller by a wire. The range covers 2 inches to 20 feet.

**Table 9:** Recent papers using advanced ultrasonic NDT.

Application	UT Type	Description	Refs
Industrial quality control	WSN-based UT	An ultrasonic-based testing system using wireless sensor networks (WSNs) is developed to monitor the thickness of compound metal sheets.	[183]
Plate monitoring at high temperatures	OFS-based LUT	A novel laser ultrasonic visualization technique using an optical fiber sensor (OFS) proposed to detect damage in a plate in a high-temperature environment.	[184]
Railway track monitoring	WSN-based UT	This work presents two novel prototypes using WSN based on a contact-less sensing mechanism.	[185]
Copper pipeline monitoring	LUT	Laser ultrasonic scanning is applied in copper pipelines to detect damage using a method based on Convolutional Neural Network (CNN) and Long Short-Term Memory Network (LSTM) networks.	[186]
Pipeline monitoring	WSN-based UT	Different testing approaches based on WSNs are presented to monitor pipeline faults remotely. WSNs are combined with ultrasonic, magnetic induction, magnetic flux leakage, and acoustic emission technologies.	[187]
Reinforced concrete monitoring	IUCT	A method using an improved ultrasonic computerized tomography method (IUCT) and compressive sampling (CS) is proposed to locate damage in concrete based on the damage’s sparse distribution.	[188]
Surface slot assessment in extreme environments	Pulsed laser	An enhanced laser-generated ultrasonic Rayleigh wave method is presented to characterize surface slots without contacting the surface. The signal-to-noise ratio of transmitted Rayleigh waves is improved using a delay-and-sum superposition technique.	[189]
Solid metal materials monitoring	ME-UT	A magnetoelectric (ME)-ultrasonic hybrid transducer and a multimodal system are developed for evaluating internal and surface defects simultaneously.	[190]
Online inspection of metal additive manufacturing	Ultra-fast laser UT	In this work, an ultra-fast laser ultrasonic imaging method is proposed as a means of efficiently inspecting metal additive manufacturing online.	[191]
Automated wrinkle identification in laminated composites	PAUT	A novel method is proposed to detect and localize wrinkles in laminated composites combining phased array ultrasonic inspection with deep learning.	[192]

**Table 10:** Overview of current advanced magnetic sensors.

Type	Characterestics	Ref
SQUID	- One of the most sensitive low-field sensors. - Josephson junctions and flux quantization are utilized in this operation. - Conventional SQUID sensors measure the magnetic flux across the pick-up coil section. The measuring principle of advanced SQUID sensors is more complex. - Sensors are configured with two pick-up coils wound in opposite directions, functioning as a magnetic gradiometer. - High sensitivity (≈10–100 ft $Hz^{-0.5}$). - Wide bandwidth ranging from DC to 10 kHz. - Broad dynamic range (>80 dB). - SQUIDs feature high-temperature superconductivity (HTS) materials.	[197]
Micro-SQUID	- Micro-SQUID sensors measure tiny magnetic crystals, nanoparticles, and molecules. - Enhanced sensitivity over conventional SQUID magnetometers. - Enhances parametric amplification and boosts flux sensitivity. - Suitable for scanning probe microscopy at low temperatures. - Frequency bandwidth up to 200 megahertz at 4 Kelvin.	[198]
Nano-SQUID	- Nano-SQUID sensors measure the magnetic properties of atoms at the quantum level. - Sensors are nano-sized detecting changes in magnetic fields. - Have the ability to detect more than magnetic flux.	[199]
OPMs	- Scalar-type quantum sensors based on the Zeeman effect for magnetic fields. - Quantum sensors with high sensitivity. - Due to their unique alkali vapor properties and their interaction with external magnetic and laser fields, they can detect magnetic fields with an unpredictably high degree of sensitivity. - Sensors have a wide dynamic range. - Cost-effective sensors.	[200]
GMI	- Directional magnetic fields can be detected. - Sensors have high resolution. - GMI sensors have been developed using electrodeposited Ni-Fe permalloy. - High sensitivity of impedance to magnetic fields. - Improvement of impedance change rate.	[201]
Fluxgate	- Fluxgate sensors provide field sensitivities in the range of a few tens of nano Tesla. - The strength of a magnetic field is measured by sensors. - The measuring principle is based on the “second harmonic principle”. - Field sensitivity is of up to 10 $pT/\sqrt{Hz}$ in the range [$10^{-10};\;10^{-3}$] T. - The frequency bandwidth is from DC to 1 kHz. - Dynamic range of 140 dB.	[202]
Hall Sensors	- Operation is based on the Hall effect. - Flux density can be measured from 100 nT. - Resolution is higher than 1 nT. - Active areas are as small as (0.1 ∗ 0.025)·$10^{-3}$ m. - Measurement of the magnetic field in a direction perpendicular to the surface. - Sensors are slabs of semiconductor material.	[203]
AMR	- Sensitivity is 1 mG to 6 G. - Frequency bandwidth is from DC to 5 MHz. - They have a limited magnetic field range, so their saturation field is relatively low. - Dynamic range is 120 dB. - Solid-state magnetic sensors are available in one, two, and three-axis versions.	[204]
GMR	- Insensitive to magnetic fields perpendicular to their sensitivity direction. - Sensor characteristics will not be disturbed if they are subjected to strong magnetic fields. - High sensitivity. - Robust probes in noisy industrial environments.	[205]

**Table 11:** Recent papers on advanced electromagnetic and magnetic technologies.

Application	NDT Type	Descriptions	Refs
Mechanical property prediction	Micro-magnetic NDT	The correlation between electromagnetic characteristics, extracted from multiple micro-magnetic NDT methods, and mechanical properties are investigated using magnetization theory and magnetic domain dynamics behavior.	[208]
Crack detection in metals	DC electromagnetic NDT	An approach to quantify cracks in moving ferromagnetic materials is proposed to characterize motion-induced eddy currents (MIECs) induced by direct current (DC) electromagnetic NDT.	[209]
Crack characterization of high-speed moving ferromagnetic material	DC electromagnetic NDT	A new DC electromagnetic NDT probe is designed, based on the drag effect for crack characterization of high-speed moving ferromagnetic materials, considering the sensitivity and strength of the detection signal.	[210]
Identification of cracks	Magnetic, optical imaging (MOI) method	Using magnetic, optical images, an improved generative adversarial network (GAN)-based crack detection method is proposed for electromagnetic NDT.	[211]
Carbon fiber reinforced polymer monitoring	Combining ECT and thermography	Based on the power loss of the micro-probe, a novel NDT method combining ECT and thermography is proposed, with a working frequency of 750 kHz.	[161]
Identification of rail foot cracking	EMAT	A novel technique is proposed for the in situ and rapid detection of cracks in the rail foot by ultrasonic B-scan imaging using a shear horizontal guided wave EMAT.	[212]
Pipeline inspection	Improved MFL	In this work, DL architectures are utilized to propose a novel real-time detection method for MFL using pattern recognition in non-destructive principles.	[213]

**Table 13:** Comparison between laser scanning and photogrammetry.

Aspect	Digital Photogrammetry	Laser Scanning
Sensing scheme	Passive	Active
Sensor type	Frame/linear sensors with perspective geometry	Point sensors with polar geometry
Ease of installation	Easy	Easy
Power level	Low	High
Acquisition of 3D coordinates	Indirect	Direct
Sampling area	Point wise sampling	Full area
Image type	Geometrical/radiometric (high quality, multispectral)	Monochromatic (low quality, single spectral)

**Table 14:** Main bridges in the world equipped with SHM systems.

No.	Bridge Name	Location	Bridge Type	Span (m)	Sensor Types Installed *	Refs
1	Sydney Harbour Bridge	Sydney, Australia	Arch	503	(2), (5)	[303]
2	Jiangyin Bridge	Jiangsu, China	Suspension	1385	(1), (2), (3), (4), (5), (6), (9), (10), (13)	[304]
3	Sutong Bridge	Jiangsu, China	Cable-stayed	1088	(1), (2), (3), (4), (5), (6), (7), (8), (9), (10), (11), (15), (17)	[305]
4	Shenzhen Western Corridor	Hong Kong, China	Cable-stayed	210	(1), (2), (3), (4), (5), (7), (8), (14), (15), (16), (17)	[306]
5	Humen Bridge	Guangdong, China	Suspension	888	(3), (6), (11), (12)	[307]
6	Lupu Bridge	Shanghai, China	Arch	550	(2), (3), (4), (12)	[308]
7	4th Qianjiang Bridge	Zhejiang, China	Arch	580	(1), (2), (3), (4), (9), (13)	[309]
8	Banghwa Bridge	Seoul, Korea	Arch	540	(1), (5), (11)	[310]
9	Stonecutters Bridge	Hong Kong, China	Cable-stayed	1018	(1), (2), (3), (4), (5), (6), (7), (8), (9), (10), (11), (14), (15), (16), (17)	[311]
10	Tongling Yangtze River Bridge	Anhui, China	Cable-stayed	432	(1), (2), (4), (11), (13)	[312]
11	Tsing Ma Bridge	Hong Kong, China	Suspension	1377	(1), (2), (3), (4), (5), (6), (7), (12), (17)	[313]
12	Great Belt Bridge	Korsør, Denmark	Suspension	1624	(2), (3), (7)	[314]
13	GOLDEN GATE BRIDGE	San Francisco, USA	Suspension	1280	(2), (4)	[315]
14	Millau Viaduct	Creissels, France	Cable-stayed	342	(11), (2), (4), (15)	[316]
15	Akashi Kaikyo	Kobe, Awaji, Japan	Suspension	1991	(1), (2), (4), (5), (6), (14)	[317]
16	Brooklyn Bridge	NYC, USA	Hybrid (suspension/ cable-stayed)	486.3	(2), (4), (5), (10)	[318]
17	I-35W Saint Anthony Falls Bridge	Minneapolis, USA	Box girder	154	(2), (3), (4), (8), (10), (15)	[319]
18	Forth Road Bridge	Scotland, UK	Suspension	1006	(6), (17)	[320,321]
19	Mackinac Bridge	Michigan, USA	Suspension	1158	(2), (3), (4)	[322]
20	Sunshine Skyway Bridge	Florida, USA	Cable-stayed	366	(1), (2), (3), (4), (6)	[323]
21	Confederation Bridge	Borden-Carleton, Canada	Box girder	250	(1), (2), (4), (5)	[324]
22	Hangzhou Bay Bridge	Jiaxing, China	Cable-stayed	325	(1), (2), (6), (7), (8)	[325]
23	Helix Bridge	Marina Bay, Singapore	Footbridge	65	(4)	[326]
24	Chesapeake Bay Bridge	Virginia, USA	Hybrid (suspension, cantilever & truss)	488	(1), (2), (6), (15)	[327]
25	Vasco da Gama Bridge	Lisboa, Portugal	Cable-stayed	420	(1), (3), (4)	[328]
26	Manhattan Bridge	New York, USA	Suspension	451	(2), (3), (4), (5), (8), (10)	[329]

* Sensor types: (1)—anemometers; (2) temperature sensors; (3) strain gauges; (4) accelerometers; (5) displacement transducers; (6) global positioning systems; (7) weigh-in-motion systems; (8) corrosion sensors; (9) elasto-magnetic sensors; (10) optic fiber sensors; (11) tiltmeters; (12) level sensors; (13) total stations; (14) barometers; (15) hygrometers; (16) pluviometers; (17) video cameras.

**Table 15:** Typical EOCs and suitable sensors for SHM systems.

EOCs	Sensory Systems
Wind	- Ultrasonic and propeller anemometers - Barometers - Visibility and precipitation sensors - Hygrometers
Temperature	- Temperature sensors - Fiber optic sensors - Thermocouples
Seismic and ship impacts	- Servotype accelerometers
Settlement	- Settlement sensors/systems - Liquid leveling system
Scouring	- Scouring sensors/systems
Corrosion	- Hygrometers - Corrosion cells - Gas concentration detectors - Temperature sensors
Highway traffic	- Dynamic weigh-in-motion stations - Static/dynamic strain gauges - High-definition video cameras
Railway traffic	- Static/dynamic strain gauges - High-definition video cameras

**Table 16:** Recent papers using model- and data-based methods in SHM systems.

Application	Method	Description	Refs
Composite laminated plate	Model-based method	This study proposed an innovative method based on a transformed form of the condensed frequency response function (CFRF) matrix using an empirical mode decomposition (EMD) algorithm to detect damage in a noisy system.	[339]
Sensors	Data-based method	This work presents a deep learning-based method, the Tsfresh long short-term memory networks (TL- STM), to determine sensor faults.	[340]
Bridge	Data-based method	This study proposed a novel deep-learning-enabled data compression and reconstruction framework based on a convolutional neural network (CNN).	[341]
Composite laminated plate	Model-based method	This article proposed a novel method using the variational mode decomposition (VMD) algorithm to construct a new set of input signals for a sensitivity-based model updating problem.	[342]
3D Truss	Model-based method	This work proposed a new optimization problem based on residual vectors and sensitivity methods using FRF in complex systems with closely spaced eigenvalues.	[343]

**Table 17:** Different types of sensors used for physical measurements in SHM systems classified by measurement type.

Property Type	Measurement Type	Sensor Type
Mechanical	Strain	Piezoresistive, Optical, Strain Gauge, Piezoelectric, Vibrating wire strain gauge
	Force	Optical, Piezoresistive, Load cells
	Fatigue	AE, Eddy currents, Strain, Ultrasonic waves
	Crack	Ultrasonic waves, GPR, AE, Thermography, Strain, Piezoelectric transductors, Hall-effect movement sensor, Vibrations
	Corrosion	Impedance, Eddy currents, AE, RFID, Strain, Magnetic waves
Ambiental	Temperature	Thermoresistive, Acoustic, Optical, Thermoelectric, Thermocouples, Thermography, RTD
	Wind	Anemometer
Kinematical	Velocity	Magnetic, Induction, Optical, Piezoelectric, Doppler effect, Electromechanical, Gyroscope
	Displacement	Gyroscope, Inductive, Ultrasonic, Capacitive, AE, Magnetic, Optical, LVDT, GPS, Optical, Resistive
	Acceleration	MEMS, Piezoresistive, Piezoelectric, Capacitive

**Table 18:** Displacement sensors.

Sensor Type	Applications	Advantages	Disadvantages
Inductive	- Crack identification in turbine blades. - Measurement of corrosion thinning is aircraft	- Low noise and interference sensitivity. - Suitable for high temperatures while remaining insensitive to environmental conditions. - High accuracy over small distances (1 mm to 150 mm).	- High-resolution measurements are affected by surface conditions.
Capacitive	- Measurement of engine door cowling gaps. - Detection of misalignments in aircraft cargo doors.	- Suitable for conductive and non-conductive materials. - High resolution and wide bandwidth.	- Highly sensitive to environmental factors. - Electrostatic charge is susceptible to frictional charges.
Gyroscope	- Measurement of turbulence-induced angular displacement of aircraft wings. - Monitoring and controlling satellite positions under hostile conditions.	- High signal-to-noise ratio. - Low power consumption.	- Drift between actual and sensed values accumulates with mechanical gyros. - Information is only relative.
Magnetic	- Measurement of ignition timing and misfire in crack shafts. - Inspection of welded steel armor plates.	- Maintenance of stability in noisy conditions. - Sensitive to low temperatures.	- Susceptible to interferences from external magnetic fields. - Only suitable for ferromagnets.
Optical	- Video monitoring of the hull deflection of a composite patrol boat. - Assessment of composite bridge deck displacements from automotive loading.	- Structure is not affected by automotive loading. - Not susceptible to electrostatic interference or stray magnetic fields.	- Light refracting at steep angles, so it cannot be bent steeply. - Textile fibers can easily be damaged.
Ultrasonic	- Investigation of wear, chipping, temperature, and breaking in tooling parts. - Inspection of aircraft wing bolts or rivets.	- Immune to external disturbances such as vibration, ambient noise, and electromagnetic radiation. - Capable of detecting minor defects from a distance.	- Damage directly below the sensors cannot be detected due to the “dead” region. - Time intensive. - Requires skilled user.
Acoustic emission	- Monitoring of seal and blade-tip rubbing in turbomachinery. - Assessment of damage to steel–concrete composite bridge decks.	- Minimizes the effect of surface roughness and geometry. - Capable of detecting crack formation due to high sensitivities.	- Noise-sensitive. - Mounting sensors on the surface may result in issues with mass loading.

**Table 19:** Velocity sensors.

Sensor Type	Applications	Advantages	Disadvantages
Magnetic induction	- Measurement of gear speed in an automotive gearbox. - Measurement of the rotational speed in gas turbine engines.	- Good sensitivity and immunity to noise. - High-speed applications have a lower output.	- Susceptible to interference from electromagnetic fields. - Installation must be perpendicular to the motion plane.
Optical	- Monitoring of automotive tire vibrations. - Monitoring of molten plastic flow during injection molding.	- Accurate and reliable. - Unaffected by surface roughness.	- Hard-to-reach parts are challenging to measure. - Require a powered light source.
Piezoelectric	- Measurement of cavitating pump vibrations. - Monitoring of seals in paper-handling machines.	- Higher bandwidth than magnetic sensors. - Reduces high-frequency signal-to-noise ratio.	- Nonlinear response at low frequencies (⩽10 Hz). - Sensor must be mounted on the structure.

**Table 20:** Acceleration sensors.

Sensor Type	Applications	Advantages	Disadvantages
Capacitive	- Monitoring of the response of aircraft wings to flutter. - Detection of acceleration of hard disk drives during writing.	- More sensitive than piezoresistive accelerometers. - Detects static acceleration.	- Must compensate for interference and drift. - Insufficient resolution and fragility.
MEMS	- Development of automotive airbag systems. - Detection of laptop vibrations and hard drive processes.	- Fast, small, and lightweight. - Cheaper than other accelerometers	- Over time, performance and specifications may degrade. - Due to their small size, they are expensive to repair.
Piezoelectric	- Measurement of exhaust system vibration. - Measurement of acceleration response of TPS panels.	- Low output noise, wide dynamic range. - Produces high voltage.	- Low bandwidth, unsuitable for testing at low frequencies. - Requires mounting the sensor to the structure, resulting in mass loading problems.
Piezoresistive	- Monitoring of ejection seat accelerations. - Measuring collision-induced acceleration of crash test dummies.	- Unaffected by electromagnetic fields. - Detects static acceleration.	- Resolution limited by resistive noise. - Designed for low- to mid-level applications.

**Table 21:** Strain sensors.

Sensor Type	Applications	Advantages	Disadvantages
Piezoresistive	- Measurement of gas turbine fan blade strains. - Measurement of blade deflections of helicopters.	- Able to measure static forces. - Simple surface mounting.	- Requires mounting the sensor to the structure. - Sensitive to temperature and external noise sources.
Optical	- Monitoring of strain in civil structures, such as buildings, bridges, dams, and pipelines. - Monitoring of ship hull strains.	- Insensitive to electromagnetic fields. - Capable of multiplexing.	- Requires fiber optic cable. - Power-dependent.

**Table 22:** Force sensors.

Sensor Type	Applications	Advantages	Disadvantages
Piezoresistive	- Impact force recording in military applications. - Measurement of wave forces on offshore oil platforms.	- High stiffness enabling machine structures to be directly inserted. - High natural frequencies, which are ideal for fast transients.	- More expensive than other types. - Possibility of nonlinear outputs.
Optical	- Automated traffic monitoring. - Measurement of window clamping force.	- Capable of multiplexing. - Suitable for high temperatures.	- Fiber optic cable is required for each sensor. - Power supply needed.

**Table 23:** Temperature sensors.

Sensor Type	Applications	Advantages	Disadvantages
Acoustic	- Measurement of the temperature inside of catalytic converters. - Measurement of temperature for engine combustion feedback control.	- Suitable for cryogenic temperatures. - Radiation-resistant.	- Noise-sensitive. - Surface-mounted sensors are required.
Optical	- Measurement of electric generator temperatures. - Monitoring of semiconductor manufacturing temperatures.	- Low electromagnetic interference. - Flexible and small for easy installation.	- Limits the maximum temperature of fiber optic cables. - Processing of data is slow.
Thermoresistive	- Temperature measurement of engine oil and coolant. - Measurement of the temperature inside HVAC systems.	- Less expensive. - Simple construction due to its small size.	- Nonlinear resistance-temperature relationship limits the range of operating temperatures. - Limited operating temperature range.
Thermoelectric	- Monitoring of exhaust gases from engines and turbines. - Heat treatment and measurement of metal processing temperatures.	- Temperature ranges higher than thermosensitive sensors. - Most inexpensive temperature sensor.	- Maximum temperature of 3100 °F. - Temperature measurements drift over time.

**Table 24:** Pressure sensors.

Sensor Type	Applications	Advantages	Disadvantages
Piezoresistive	- Measurement of engine combustion chamber pressure. - Measurement of each component’s inlet and outlet pressure.	- Capable of measuring static and dynamic pressures. - Can be relied upon in varying environmental conditions.	- Pressure increases may cause the transducer to become nonlinear. - Electrical noise may result.

**Table 25:** Sensor specifications based on maximum operating temperature.

Sensor	Application	Maximum Operating Temperature, °C	Year of Development	Refs
Capacitive sensor based on interdigitated electrodes	Acceleration measurement	60	2019	[395]
Electrostatic MEMs sensor based on CMOS	Acceleration measurement	175	2020	[396]
UV irradiated Type I FBG sensor	Axial vibration measurement	70	2018	[397,398]
Planar coil eddy current sensor	Displacement measurement	350	2019	[399]
Polyimide-coated pulse UV irradiated Type II FBG sensor	Strain measurement	450	2012	[400]
MEMs resonator array made of piezoelectric material	In-situ defect detection	500	2019	[401]
Wireless capacitive sensor using LTCC technology	Pressure measurement	600	2013	[402]
Meander patterned eddy current sensor	Damage assessment of boiler component	730	2009	[403]
Fs-IR induced FBG sensor	Pressure measurement	800	2010	[404]
Fs-IR induced FBG sensor	Temperature and strain measurement	1000	2010, 2020	[398]
Gas sensor using LTCC technology for micro-hot plates	Gas monitoring	850	2018, 2020	[405]
Proximity sensor based on LTCC technology	Proximity measurements	1000	2019	[406]
Photonic Crystal Fiber based Fabry Perot strain sensor	Strain measurement	1100	2021	[407]
Ultrasonic transducer made of lithium niobate	In-situ defect detection	1100	2019	[408]

**Table 26:** Review of some recent papers on the application of advanced sensors in SHM.

Refs	Year	Model	Sensor Type	Description
Tennyson et al. [422]	2001	Bridge	FBG	This paper described the development and application of FBG sensors for monitoring bridge structures in Canada.
Ma and Asundi [423]	2001	Aluminum specimen	Fiber optic polarimetric sensor (FOPS) & fiber optic curvature sensor (FOCS)	An FOPS and a FOCS were theoretically and experimentally analyzed for global SHM.
Lee et al. [424]	2002	Aerospace structures	Extrinsic Fabry-Perot Interferometric fiber optic strain sensors (EFPI-FOSS)	This paper presented experimental results on the thermomechanical behavior of EFPI-FOSS.
Baldwin et al. [425]	2002	British Trimaran Research Vessel (RV) Triton	FOS	This paper described the installation and testing of a large-scale FOS network.
Leng and Asundi [426]	2003	CFRP composite laminates	EFPI & FBG	Sensors were employed to monitor the curing process of the model with and without damage simultaneously.
Qing et al. [427]	2005	Aerospace vehicles and structures	FOS	A hybrid diagnostic system was developed for quick non-destructive evaluation and long-term health monitoring.
Leng et al. [428]	2006	Concrete cylinders	Protected EFPI & FBG	Two sensor protection systems were developed in this work.
Li and Wu [429]	2007	Steel and reinforced concrete (RC) structures	Long-gauge FBG	This paper summarized the manufacturing method of the developed sensors and verified their performance.
Zagrai et al. [430]	2010	Space structures	Piezoelectric Wafer	This paper explored specifics of SHM applied to space systems and satellites.
Rice et al. [431]	2010	Cable-stayed bridge	Imote2 smart sensor	A flexible wireless smart sensor framework was developed for autonomous SHM.
Fraser et al. [432]	2010	Reinforced concrete highway bridge	An accelerometer sensor array and an integrated camera	A bridge monitoring TestBed was developed for sensor networks and related decision-support technologies.
Guo et al. [433]	2011	Air Platforms	FOS	Some recommendations were provided on the implementation and integration of FBG sensors into an SHM system.
Bocca et al. [434]	2011	Wooden model bridge	3-axis digital accelerometer and temperature and humidity sensors	This article introduced a time-synchronized and configurable wireless sensor network for SHM.
Laflamme et al. [435]	2013	Wood and concrete specimens	Capacitive sensor	A capacitive sensor with tailored mechanical and electrical properties was presented.
Song et al. [436]	2014	Cantilever beam	Virtual visual sensors	The authors evaluated a proof-of-concept application of virtual visual sensors to well-known engineering problems.
Feng and Feng [437]	2016	Three-story frame structure	Vision-based multipoint displacement sensors	A novel noncontact vision sensor was developed for simultaneous measurement of structural displacements at multiple points using one camera.
Feng and Feng [438]	2017	Bridge	Advanced non-contact vision-based sensors	This study validated the potentials of the vision displacement sensors for cost-effective SHM.
Chilelli et al. [439]	2019	Metal Structures	FBG	FBG sensors embedded in an aluminum matrix were investigated with a focus on detecting crack initiation and growth.
Huan et al. [440]	2019	Thickness-poled piezoelectric half-rings	Omni-directional Shear horizontal wave transducer	A practical transducer was developed.
Loubet et al. [441]	2019	Structures in harsh environments	Battery-free wireless sensor	This paper addressed the concept of a wirelessly powered sensor for cyber-physical systems.
Li et al. [442]	2020	Joint member	High precision FBG displacement sensor	In this paper, an FBG displacement sensor with an embedded spring was developed to monitor structural displacement variation even at minimal ranges.
Giurgiutiu [443]	2020	Aerospace composite structures	FBG and piezoelectric wafer active sensors (PWAS)	This chapter presented the major sensor classes used in SHM practice with a focus on advanced sensors.
Gómez et al. [444]	2020	Tunnel lining	Distributed FOS system	This paper addressed the implementation of a distributed FOS system to the TMB L-9 metro tunnel in Barcelona for SHM purposes.
Ghosh et al. [445]	2020	Concrete beam members	Piezoceramic sensor	This work presented a cost-effective ‘Industry 4.0’solution for real-time SHM.
Maraveas and Bartzanas [446]	2021	Agricultural structures	Electrochemical, ultrasonic, wireless, FOS, and piezoelectric sensors	The cost–benefits of each type of sensor and utility in a farm environment were explored in this review.
Di Nuzzo et al. [447]	2021	Steel structure	Low-cost wireless sensor	This work proposed a sensor node specifically designed to support modal analysis over extended periods with long-range connectivity at low power consumption.
Aulakh and Bhalla [448]	2021	Beam	Piezoelectric sensors	This paper aimed to evaluate the piezoelectric sensors for modal response measurement, modal parameter identification, and SHM of 3D structures, especially using torsional modes.
Mieloszyk et al. [449]	2021	Fast patrol boat	FBG	An application of embedded FBG sensor arrays was presented for evaluating complex composite structures.
Barsocchi et al. [450]	2021	Historic masonry towers	Micro-electromechanical sensors	This paper discussed a monitoring system made of the sensors connected through a wireless network.
Braunfelds et al. [451]	2021	Road infrastructure	FBG	This article focused on the research of the FBG optical temperature and strain sensor applications in road SHM.
Komarizadehasl et al. [452]	2022	Bridge	Low-Cost wireless sensors	This work presented a new low-cost triaxial accelerometer based on Arduino technology.
Giannakeas et al. [453]	2022	Aircraft fuselage	Piezoelectric transducers	A bottom-up framework was presented to estimate the initial investment cost and the added weight associated with the integration of an SHM system to an aircraft.
Zini et al. [454]	2022	Historical city gates	Accelerometers	This paper reported on a pilot project for long-term SHM of historical city gates.
Pittella et al. [455]	2022	Concrete beams	A diffused sensing element and a split ring resonator network	This work aimed to propose two different and integrated sensors for the SHM of concrete beams.
Hao et al. [456]	2022	Large-scale civil engineering infrastructures	Energy-aware versatile wireless sensor	A sensor network configuration optimization approach was proposed to design informative and energy-efficient wireless sensor networks.
Roopa and Hunashyal [457]	2022	Beam and column	Cement-based nanocomposite sensors	This paper presented the development and implementation of cement-based nanocomposite sensors for SHM applications.
Franchi et al. [458]	2023	Building	5G-Based Network	Some preliminary results from an advanced SHM system were shown.
Figueiredo et al. [459]	2023	Bridge	Smartphone	A smartphone application was developed to apply on SHM systems to assess their condition after a catastrophic event.

**Table 27:** Signal processing methods used for SHM.

Technique	Strengths	Shortcomings
KF	- Reasonable signal-noise ratio - Identification of time changes effectively	- Calibration of parameters is necessary - Time-consuming - Tracking accuracy and convergence speed are limited
STS	- Linear model - Simple implementation	- Noise-sensitive - Only suitable for linear systems
FFT	- Nonlinear model - Is capable of modeling linear and nonlinear systems - Simple implementation	- Unsuitable for complex systems - Requires calibration to determine order - Noise-sensitive - Representation in only the frequency domain
MUSIC	- High frequency resolution - Estimation of closely spaced modes	- Time-consuming
SFFT	- Simple implementation - Representation in the time-frequency domain	- Requires a large number of samples - Insufficient time-frequency resolution - Inapplicable to nonlinear signals and transients
ST	- High time-frequency resolution - Spectrum can be localized in the time domain	- Time-consuming - Needs calibration
FST	- Time-saving - Provides a good time-frequency resolution - Time-domain localization of spectrum	- Suitability for SHM applications is still under investigation
WT	- High time-frequency resolution - Good signal-to-noise ratio - Mother wavelets can be used for different applications	- Spectral leakage - Needs several levels of decomposition
HHT	- Good time-frequency resolution - High signal-to-noise ratio - Adaptive method - Simple implementation	- Mode-mixing - Requires calibration
BSS	- Good signal-noise ratio - Estimation of closely spaced modes - Ability to separate frequencies accurately	- Require calibration - Analyzing nonlinear and transient signals is difficult
Cohen’s class	- Effectiveness in computation - High resolution in time-frequency domain - Observation of closely spaced modes	- Distribution function characteristics depend on kernel selection

**Table 28:** Recent papers on DL in SHM systems.

DL Method	Model	Descriptions	Refs
CNN	Plate model	This work presents a hierarchical deep convolutional regression framework to solve the impact source localization problem based on CNN using acoustic emission signals. It is verified on a simple homogeneous plate and a complex inhomogeneous plate.	[472]
LSTM	Bridge	This article proposes an LSTM-based real-time approach using an unsupervised LSTM prediction network for detection.	[473]
CNN	Grandstand simulation	This work proposes a novel system using CNNs to fuse feature extraction and classification blocks into a single and compact learning body.	[474]
GAN	Concrete	A balanced semi-supervised GAN (BSS-GAN) was proposed using the semi-supervised learning concept and balanced batch sampling in training to solve low-data and imbalanced class problems.	[475]

## Data Availability

Not applicable.
